# VDR-Spermidine Axis Protects Against Age-Related Granulosa Cell Dysfunction and Follicular Decline via DNMTs-Mediated p53 Methylation

**DOI:** 10.7150/ijbs.128631

**Published:** 2026-05-18

**Authors:** Haiyun Chen, Qinghe Geng, Qiuyi Wang, Yuanqin Li, Luxi Shangguan, Zhengquan Zhu, Jingwen Zhang, Shuan Wang, Xiaotian Chen, Shanmei Shen, Yanting Wen, Daojuan Wang, Yong Wang

**Affiliations:** 1 Department of Clinical Nutrition, Nanjing Drum Tower Hospital, Affiliated Hospital of Medical School, Nanjing University, Nanjing 210008, Jiangsu, PR China.; 2 State Key Laboratory of Analytical Chemistry for Life Science & Jiangsu Key Laboratory of Molecular Medicine, Medical School, Nanjing University, Nanjing, China.; 3 Key Laboratory of Clinical Research of Osteoporosis, Xuzhou Medical University, Xuzhou 221300, China.; 4 Central Lab, Pizhou Hospital, Xuzhou Medical University, Xuzhou 221300, China.; 5 Liangzhu Laboratory, School of Medicine, Zhejiang University, Hangzhou, China.; 6 Department of Endocrinology, Endocrine and Metabolic Disease Medical Center, Nanjing Drum Tower Hospital Clinical College of Nanjing University of Chinese Medicine.; 7 Department of Pain, Nanjing Drum Tower Hospital, Affiliated Hospital of Medical School, Nanjing University, Nanjing 210008, China.

**Keywords:** ovarian aging, spermidine, DNMTs, methylation

## Abstract

Ovarian aging, marked by a decline in follicle quantity and quality, is a complex process whose underlying mechanisms remain elusive. Here, we identify the vitamin D receptor (Vdr) as a key anti-aging transcription factor whose expression in granulosa cells (GCs) declines with age. Using GCs-specific *Vdr* knockout (cVKO) mice and a *Vdr*-knockout (VKO) human granulosa-like cell line, we demonstrate that loss of *Vdr* triggers GCs aging and disrupts ovarian function. Integrated transcriptomic and metabolomic analyses from VKO and WT cells revealed that *Vdr* loss downregulates the *de novo* spermidine (SPD) biosynthesis by directly suppressing the transcription of ornithine decarboxylase (*ODC1*). This led to SPD depletion, which in turn inhibited DNA methyltransferase (DNMTs) activity, resulting in hypomethylation of the p53 promoter and activation of the p53/p21 pathway. Crucially, supplementation with either SPD or its upstream methyl donor S-adenosylmethionine (SAM) rescued cVKO and VKO cell aging, improved hormonal profiles and promoted follicular development in cVKO mice. Furthermore, both supplements effectively delayed ovarian aging and improved fertility in naturally aged mice. Our study unveils the Vdr-spermidine-DNMTs axis as a fundamental mechanism safeguarding against ovarian aging, highlighting SPD and SAM as promising therapeutic agents for age-related female infertility.

## Introduction

The fundamental cause of female reproductive aging is ovarian aging. However, in the current context where childbearing age is widely delayed due to various social factors, and environmental factors may lead to the premature onset of ovarian aging during a woman's lifespan, the decline in fertility caused by ovarian aging has become a major source of emotional distress for many women with reproductive intentions^1^. In addition to impaired fertility, numerous studies have also reported the adverse effects of ovarian aging on various non-reproductive functions in women, including an increased risk of age-related chronic health issues, such as osteoporosis, neurodegenerative diseases, and cardiovascular diseases^2^. Therefore, ovarian aging, combined with women's longer life expectancy compared to men, may be one of the potential drivers behind the so-called mortality-morbidity paradox that is, meaning that women generally live longer but experience a relatively lower quality of life^3^. This reality underscores an urgent need to improve strategies for addressing ovarian aging, both to preserve fertility and to enhance the overall health and quality of life for affected women.

Ovarian aging is characterized by the decline of ovarian function-primarily due to the diminishing quantity and quality of the ovarian follicle pool-which results in reduced fertility, hormonal imbalances, and ultimately menopause^4^-^6^. Within follicles, granulosa cells (GCs) are essential somatic components that provide critical support for oocyte development and follicle maturation^7^-^9^. Oocyte-granulosa cell interactions regulate follicular growth and atresia^10^. As transcriptomic and metabolomic changes occur in GCs during aging, the altered GCs metabolism directly influences the health and competency of maturing oocytes^8^, ^11^-^13^. The functional integrity of GCs is therefore paramount for female reproductive health, and their aging is considered a key contributor to age-related oocyte meiotic defects and aneuploidy^11^. Follicular impairment is a primary cause of premature ovarian insufficiency^14^-^16^. Extensive research has been conducted to determine the mechanisms underlying ovarian aging. So far, multiple transcription factors, metabolic pathways and epigenetic changes have been implicated in the regulation of ovarian aging. Given the critical roles of specific transcription factors (e.g., FOXP1, CEBPD) and metabolites (e.g., spermidine, cholesterol, NAD^+^) in ovarian aging, modulating these key factors and their associated pathways presents a promising strategy to counteract age-related ovarian decline^5^, ^11^, ^12^, ^17^, ^18^. However, it remains elusive whether (and how) the crosstalk among these transcriptional, metabolic, and epigenetic regulations is coordinated.

Vitamin D, through its receptor VDR, is essential for regulating calcium and phosphate metabolism and maintaining a properly mineralized skeleton^19^. Studies have shown that vitamin D-deficient mice exhibit shortened lifespans and develop organ abnormalities characteristic of premature aging^20^. In line with this, vitamin D deficiency is considered a driver of accelerated senescence in multiple tissues, including lung, bone, skin, and muscle, contributing to their functional decline^21^. VDR, a member of the steroid hormone receptor superfamily, mediates these effects by forming heterodimers with the retinoid X receptor (RXR) upon ligand binding, thereby initiating transcription of target genes.

Polyamines, such as spermidine (SPD), are ubiquitous polycations with demonstrated anti-aging properties across model organisms^22^, ^23^. SPD levels decline with age in multiple tissues, and its supplementation can extend lifespan and improve organ function, including in the ovary^18^, ^24^. Intriguingly, SPD biosynthesis is metabolically linked to the methyl donor S-adenosylmethionine (SAM), which is decarboxylated to provide an aminopropyl group for SPD formation. This SAM pool is essential for DNA methyltransferases (DNMTs), which maintain DNA methylation patterns crucial for gene silencing and genomic stability^25^, ^26^. Notably, the decarboxylated form of SAM (dcSAM) is a potent inhibitor of DNMTs, suggesting that polyamine metabolism and epigenetic regulation are intricately connected^25^. Age-associated dysregulation of DNA methylation, including hypomethylation of promoters for aging-related genes like p53, is a well-established feature of cellular aging^27^, ^28^.

In the present study, we analyzed the transcriptomes of ovaries from young and old mice and identified the transcription factor Vdr as a pivotal regulator in preventing GCs aging. Beyond its classical role in regulation of calcium homeostasis, Vdr has emerged as a regulator of cell proliferation, metabolism and aging in various tissues^29^-^31^. Here, we found that Vdr was predominantly expressed in ovarian GCs, where its expression decreased with advancing age. SPD levels decreased in aged ovaries and supplementation of SPD could improve the age-associated decline of ovarian function^18^, ^32^. Despite increasing evidence that both of polyamine metabolism and epigenetic regulation contribute to regulation of cellular aging, whether and how these pathways are functionally integrated in ovarian GCs remains largely unknown. Vdr has been implicated in the regulation of cellular metabolism, mitochondrial function, and aging processes in multiple tissues^33^-^35^. However, the role of Vdr in GCs aging and ovarian functional decline has not been fully elucidated. It has been reported that vitamin D supplementation promotes the synthesis of polyamines in the intestine^36^. Given the close metabolic linkage between SPD biosynthesis and S-adenosylmethionine dependent DNA methylation, we hypothesized that Vdr coordinate polyamine metabolism and epigenetic regulation to preserve GCs function during ovarian aging. In this study, we therefore investigated the role of Vdr in GCs and explored its potential involvement in regulating SPD metabolism, DNA methylation, and age-associated ovarian dysfunction.

## Results

### Loss of *Vdr* accelerates GCs aging

**Figure 1:**
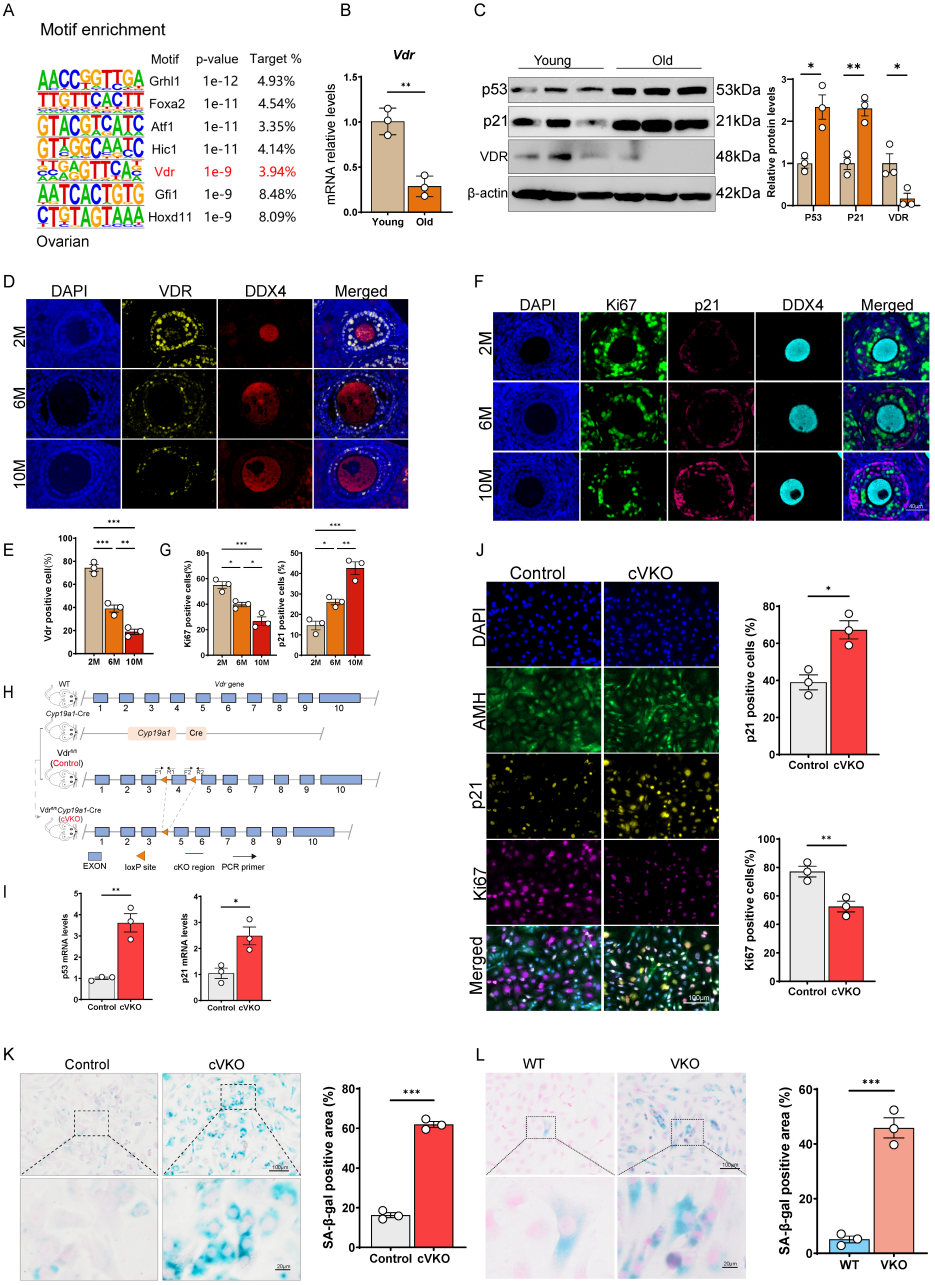
** Loss of *Vdr* induces aging of GCs.** (A) Motif enrichment analysis of differential genes from aged ovaries compared to young controls (GSE154890). (B) *Vdr* mRNA levels by real-time RT-PCR relative to *β-actin* mRNA in ovaries from young and aged mice (n = 3). (C) Western blots of ovary proteins showing p53, p21, and Vdr expression. Densitometric analysis was used to assess protein levels relative to β-actin (n = 3). (D) Immunofluorescence showing ovary staining for Vdr and DDX4, with nuclei staining for DAPI. (E) Analysis for Vdr-positive cells in the ovary. (F) Immunofluorescence showing ovary staining for Ki67, p21, and DDX4, with nuclei staining for DAPI. (G) Analysis for Ki67 and p21 positive cells in ovaries. (H) Schematic diagram of gene editing in cVKO mice. (I) *p53* and *p21* mRNA levels by real-time RT-PCR relative to *β-actin* mRNA in ovaries from control and cVKO mice. (J) Immunofluorescence showing ovaries staining for Ki67, p21, and Amh, with nuclei staining for DAPI. And analysis for Ki67 and p21 positive cells in ovaries (n = 3). (K) SA-β-gal staining for granulosa cells of control and cVKO mice, and analysis for SA-β-gal positive cells (n = 3). (L) SA-β-gal staining for WT and VKO KGN cells, and analysis for SA-β-gal positive areas (n = 3). Significance was determined using unpaired Student's t-test in B, C, I, J, K, L and one-way ANOVA followed by Dunnett's multiple comparison test in E and F. *P < 0.05, **P < 0.01 and ***P < 0.005.

To investigate the transcriptional and metabolic alterations associated with ovarian aging, we employed HOMER to predict transcription factors potentially driving gene expression changes in aged ovarian tissue. Our analysis indicated that multiple transcription factors may be involved in this regulatory process (Figure 1A). Quantitative PCR results showed that *Vdr* mRNA level was significantly downregulated in aged ovaries (Figure 1B and S1A). Immunoblotting confirmed that Vdr was significantly downregulated, while the aging markers p21 and p53 were elevated in aged ovarian tissue (Figure 1C). Using mIHC with DDX4 as an oocyte marker (DDX4 was specifically expressed in the cytoplasm of oocytes, whereas no signal was detected in granulosa or theca cells^37^), we found that Vdr expression was predominantly localized to GCs and exhibited a progressive, age-dependent decrease (Figure 1D and 1E). We also observed an age-associated increase in p21 and a concomitant decrease in the proliferation marker Ki67 within GCs (Figure 1F and 1G). To further clarify whether Vdr is downregulated in GCs with increasing age, we isolated primary GCs from young and middle-aged female mice (6-month-old) and performed western blot analysis. The results showed that Vdr expression was downregulated with age, while p53 and p21 expression were upregulated (Figure S1B). Based on these findings, we hypothesized that the loss of *Vdr* drives GCs aging by causing transcriptional changes in aged mice. To test this, we generated a granulosa cell-specific *Vdr* knockout (conditional VKO, cVKO) mouse model using the *Cre-loxP* system (Figure 1H and S1C) and a VDR-knockout (VKO) KGN cell line via CRISPR-Cas9 (Figure S1D-S1F). Primary GCs were isolated from cVKO and control mice, with successful isolation validated by high Amh expression (Figure 1J). Quantitative PCR analysis demonstrated that *Vdr* knockout led to significant upregulation of p53 and p21 mRNA levels in GCs (Figure 1I). Furthermore, mIHC staining of GCs from cVKO mice showed increased p21 and decreased Ki67 levels, supporting a potential anti-aging role for Vdr (Figure 1J). Immunoblotting analysis of isolated GCs from cVKO and Control mice further confirmed that p21 expression levels were increased in GCs from cVKO mice, while Amh and Ki67 expression levels were decreased in GCs from cVKO mice (Figure S1I). We assessed the impact of *Vdr* knockout on cellular senescence using SA-β-gal staining. The results showed that *Vdr* knockout accelerates the aging of both primary GCs and KGN cells (Figure 1K and 1L).

To investigate whether the ligand of Vdr, vitamin D, is downregulated with increasing age, we analyzed the levels of serum vitamin D and active vitamin D (1,25(OH)_2_D). The results showed that compared with young female mice aged 2 months, there was no significant difference in vitamin D levels in 10-month-old female mice, while 1,25(OH)_2_D levels showed only a slight decrease in the 10-month-old mice (Figure S1G). To further clarify the relationship between 1,25(OH)_2_D and Vdr expression, we treated KGN cells with 1,25(OH)_2_D and detected Vdr levels by western blot. The results indicated that 1,25(OH)_2_D treatment did not promote Vdr expression (Figure S1H). In summary, aging ovarian tissue exhibits a marked decline in the proliferative capacity alongside a significant increase in cellular aging of GCs. These changes coincide with the downregulation of Vdr, which is predominantly expressed in GCs. Genetic ablation of *Vdr* further accelerates GCs aging, underscoring its critical role in mitigating the ovarian aging process. However, the change of Vdr in aged ovaries are not associated with serum vitamin D levels.

### Loss of *Vdr* promotes mitochondrial dysfunction in GCs

**Figure 2:**
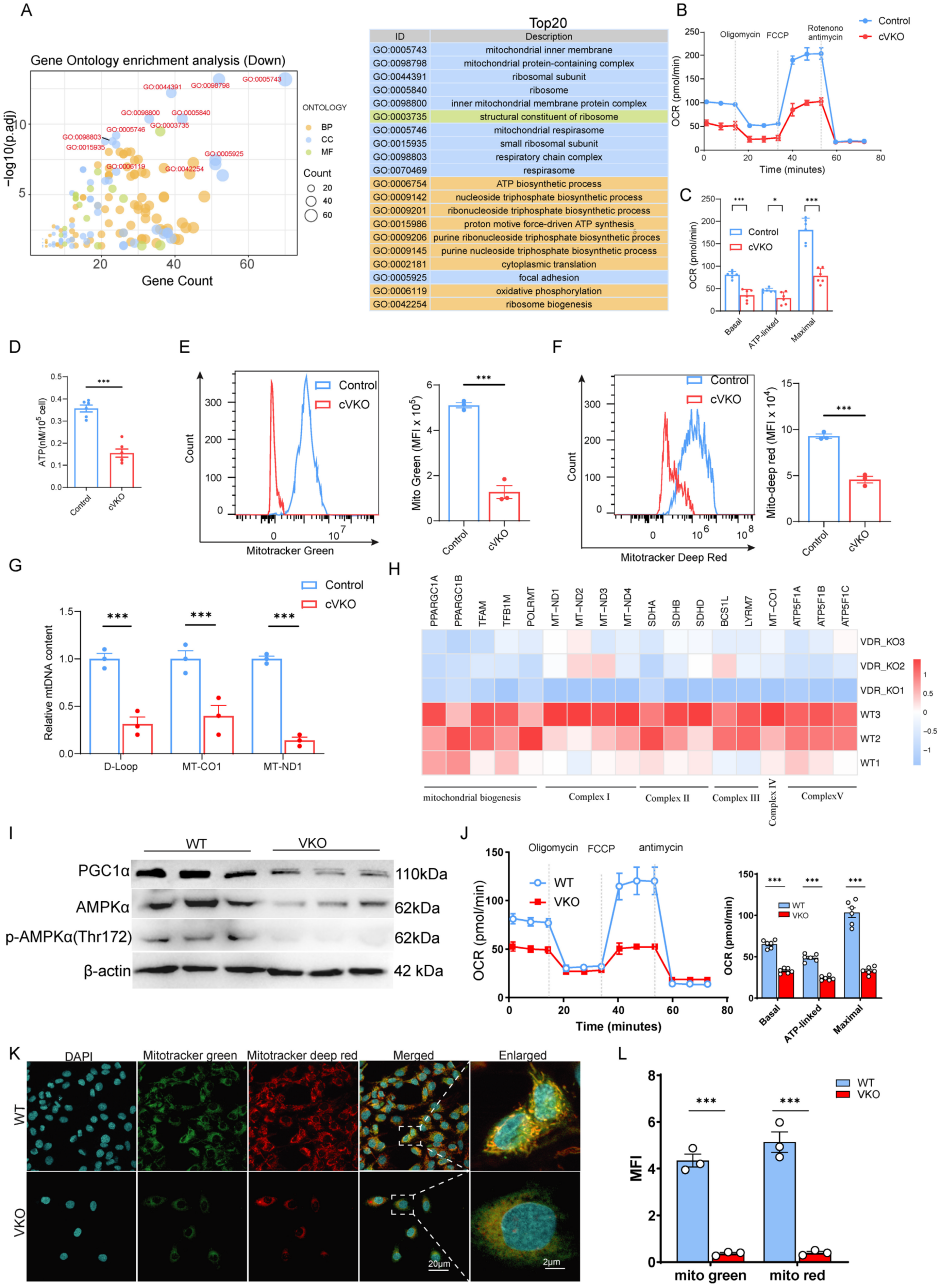
** Loss of *Vdr* downregulated the quantity and quality of mitochondria in granulosa cells.** (A) Gene Ontology analysis of differentially expressed genes from aged hGCs compared to young controls. (B-C) Measurement of oxygen consumption rate (OCR) in indicated GCs. After measurement of basal OCR, oligomycin, FCCP and rotenone+antimycin A were sequentially added, and the alterations in OCR were recorded and normalized to cell number. Quantification of the basal OCR, ATP-coupled OCR and maximal OCR is shown (n = 6). (D) ATP content quantification in indicated GCs (n = 6). (E-F) Flow cytometric analysis of mitochondrial status in indicated GCs. (n = 3 biological replicates per group). (G) Mitochondrial mass determined by mtDNA/nuclear DNA (nDNA) ratio in indicated GCs (n = 3 biological replicates per group). (H) The heatmap shows the mitochondria-related gene changes in VKO cells compared with WT cells. (I) Immunoblot analysis of mitochondria biogenesis-related proteins in WT and VKO cells. β-actin was used as a loading control. (J) The representative curves of OCR in WT and VKO cells, and quantification of the basal OCR, ATP-coupled OCR and maximal OCR are shown (n = 6). (K) Confocal microscopy analysis of mitotracker green and mitotracker deep red in WT and VKO cells. (L) The fluorescence intensity of mitotracker Green and mitotracker Deep Red was quantified using ImageJ (n = 3). Significance was determined using an unpaired Student's t-test. *P < 0.05, **P < 0.01 and ***P < 0.005.

To investigate the molecular events in aging granulosa cells (GCs), we employed Gene Ontology (GO) analysis on RNA-seq data from natural aging GCs (GSE175836). The results indicated that the top-ranked terms were primarily associated with mitochondrial function (Figure 2A). We analyzed the expression levels of genes related to mitochondrial function, and the results showed that the levels of genes related to the electron transport chain (ETC) and tricarboxylic acid cycle (TCA cycle) were generally downregulated (Figure S2A). Similarly, Gene Set Enrichment Analysis (GSEA) revealed pronounced mitochondrial abnormalities in KGN cells following *Vdr* loss-induced aging (Figure S2B). To experimentally validate these mitochondrial abnormalities, we measured mitochondrial oxidative phosphorylation (OXPHOS) via the cellular oxygen consumption rate (OCR). The results showed that GCs from cVKO mice had impaired mitochondrial respiration, with significant reductions in basal, ATP-linked, and maximal respiratory capacity compared to controls (Figure 2B and 2C).

The assessment of intracellular ATP levels provided further evidence of compromised mitochondrial function (Figure 2D). Quantification of mitochondrial number and membrane potential by fluorescence-based flow cytometry revealed that *Vdr* loss caused a reduction in both mitochondrial quantity and quality (Figure 2E and 2F). Additionally, an apparent decline in mtDNA content was also observed in the GCs of cVKO mice (Figure 2G). VKO also showed reduced mRNA levels of the mitochondrial biogenesis and mitochondrial complex-related genes (Figure 2H). Mitochondrial biosynthesis is predominantly regulated by the PGC-1α signaling pathway, which is activated upstream by AMPK. Immunoblotting results revealed that, compared to the WT group, VKO cells exhibited a significant decrease in the phosphorylation of AMPK at Thr172 (p-AMPK) and in the protein levels of PGC-1α (Figure 2I) indicating a decreased quantity of mitochondria. We further analyzed the expression of mitochondrial structural proteins as well as proteins involved in mitochondrial membrane fusion and fission. The results showed that loss of *Vdr* in GCs from cVKO mice downregulated the expression levels of the mitochondrial fusion-related protein Mfn2 and the mitochondrial structure-related proteins Atp5a, mt-co1, Sdhb, and Ndufb8, whereas it upregulated the expression level of the mitochondrial fission-related protein Drp1 (Figure S2C). These results indicate that loss of *Vdr* impairs mitochondrial fusion, enhances mitochondrial fission, and disrupts the integrity of mitochondrial OXPHOS. VKO cells also showed severe respiratory chain deficiency, as indicated by the disruption of mitochondrial respiration during OCR measurements (Figure 2J). Likewise, assessment with fluorescent probes revealed a decrease in both mitochondrial content and function in VKO cells (Figure 2K and 2L). Overall, these results demonstrate that *Vdr* loss impairs mitochondrial biogenesis, respiration, and quantity in granulosa cells.

### Loss of *Vdr* disrupts polyamine metabolism

**Figure 3:**
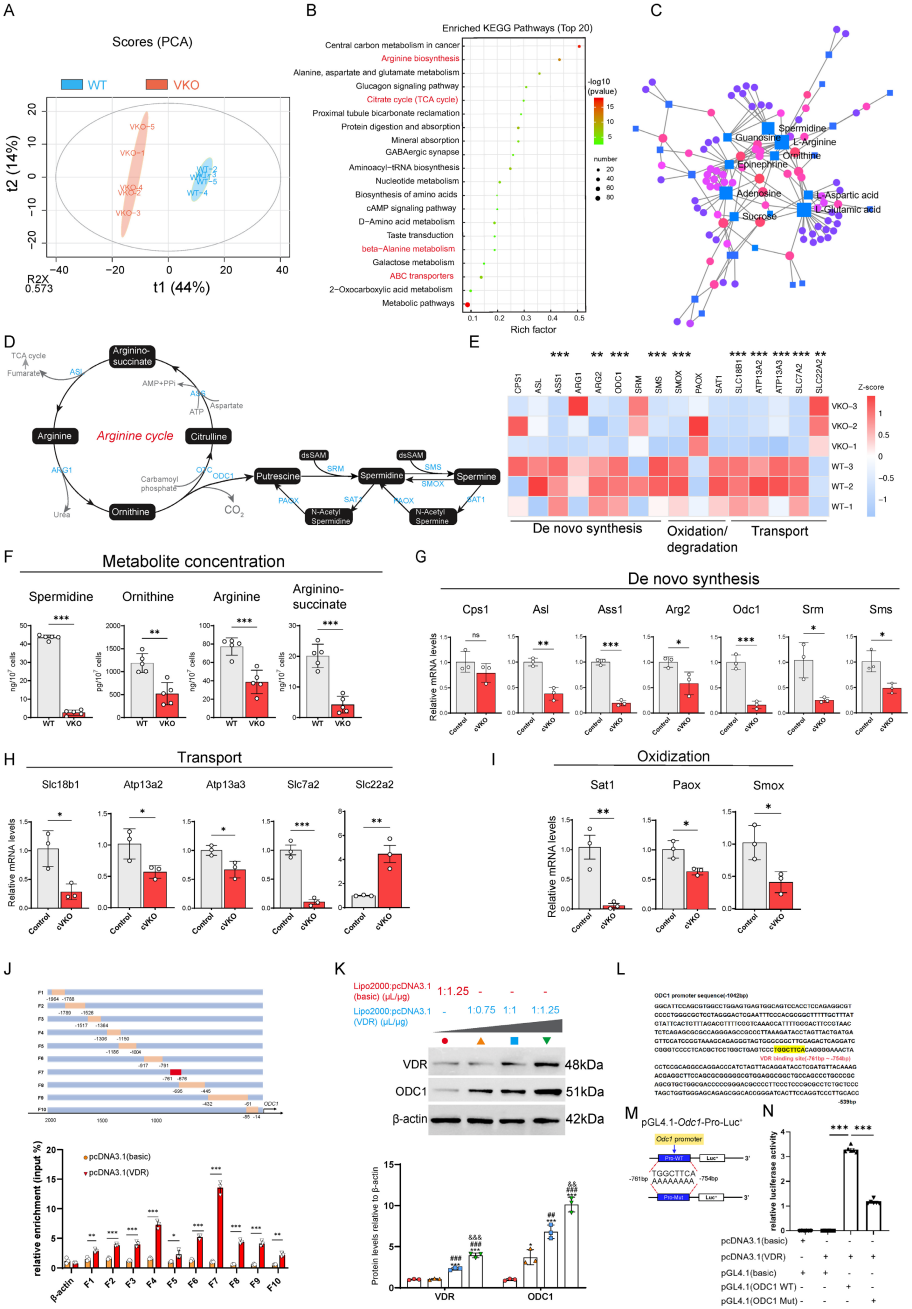
** Loss of *Vdr* disrupts polyamine metabolism.** (A) Principal-component (PC) analysis of metabolomics data from VKO and WT cells. Blue circles represent samples from the WT group, and red circles represent samples from the VKO group. (B) KEGG enrichment analysis of differential metabolites in VKO cells compared to those from the WT group. (C) Association analysis between differential metabolites and differentially expressed genes. (D) Schematic presentation of the arginine cycle. (E) Heatmap showing levels of genes related to polyamine synthesis. (F) Concentration of metabolites in VKO cells compared to the WT group. Values are the means ± SEM of six determinations per group. **P < 0.01, ***P < 0.001 compared with the WT group, unpaired Student's t-test (n=5). (G-I) mRNA levels of genes related to *de novo* synthesis, transport and oxidation of polyamine by real-time RT-PCR relative to *β-actin* mRNA in GCs of control and cVKO mice. Values are the means ± SEM of three determinations per group. *P < 0.05, **P < 0.01, ***P < 0.001 compared with the control group, unpaired Student's t-test (n=3). (J) Schematic diagram of design for truncated primers on *ODC1* promoter (upper). And ChIP assays of chromatin prepared from KGN cells with or without Vdr transfected (lower). Values are the means ± SEM (n=3). *P < 0.05, **P < 0.01, ***P < 0.001 compared with the control group, unpaired Student's t-test. (K) Western blots of KGN cells showing VDR and ODC1 expression. Densitometric analysis was used to assess protein levels relative to β-actin. Values are the means ± SEM (n=3). *P < 0.05, ***P < 0.001 compared with basic-transfected group; ##P < 0.05, ###P < 0.001 compared with the ratio of Lipo2000 relative to plasmid of 1μL/0.75μg GATA4-transfected group; &&P < 0.01, &&&P < 0.001 compared with the ratio of Lipo2000 relative to plasmid of 1μL/1μg GATA4-transfected group, one-way ANOVA. (L) The binding site of Vdr (highlighted with yellow) in the *ODC1* promoter region. (M) Schematic diagram of pGL4.1-*ODC1* promoter reporter plasmid and pGL4.1-*ODC1* promoter reporter plasmid with binding site deletion. (N) Luciferase activity assay. Statistical analysis was performed with one-way ANOVA. Values are means ± SEM (n=6). ***P < 0.001.

Given the particular relevance of Vdr for cell metabolism and mitochondrial deficiency, we performed transcriptome and metabolome analysis using WT and VKO cells. Principal component analysis of the metabolome data showed a clear separation between WT and VKO samples, with the five biological replicates forming distinct clusters within each group (Figure 3A). Heatmap analysis of differential metabolites revealed pronounced alterations in the metabolome profiles of VKO cells compared to WT cells (Figure S3A). Correspondingly, distinct differences in transcriptional patterns were also observed (Figure S3B). Additionally, the volcano plot revealed numerous differentially expressed metabolites in the VKO group compared to the WT group (Figure S3C). Based on our metabolome data, KEGG enrichment analysis revealed the top 20 pathways that were most significantly altered in the VKO group relative to the WT controls (Figure 3B). Of these, one pathway was significantly upregulated, while nineteen were downregulated, as quantified by the differential abundance score (Figure 3B and S3D). The results further suggest that *Vdr* knockout downregulates the citrate cycle (TCA cycle) in cells (Figure 3B), indicating a rewiring of cellular metabolism. After analyzing the metabolites involved in these pathways, we found that SPD, an anti-aging molecule, was present in both Arginine biosynthesis, beta-Alanine metabolism and ABC transporters as a downregulated metabolite induced by *Vdr* knockout (Figure 3B and Supplementary Table S3). The integration of transcriptome and metabolome data identified arginine, ornithine, and SPD as the central components of the gene regulatory network (Figure 3C). Since the de novo synthesis of polyamines commences with arginine (Figure 3D), we observed a coordinated downregulation of genes responsible for polyamine synthesis, transport, and oxidation/degradation in VKO cells compared with WT cells from our RNAseq dataset (Figure 3E). Metabolite analysis revealed a significant downregulation in the concentrations of key molecules involved in the de novo polyamine synthesis pathway-including the arginine precursors argininosuccinate, arginine, ornithine, and spermidine-in VKO cells (Figure 3F). Correspondingly, quantitative PCR and immunoblotting confirmed that the mRNA and protein levels of these genes were also significantly decreased in GCs in cVKO mice (Figure 3G-I and S3F). Based on these results, we propose that Vdr modulates aging through the regulation of polyamine biosynthesis.

To further investigate the mechanistic basis of this regulation, we used JASPAR (https://jaspar.genereg.net/) to scan the promoter regions of key polyamine biosynthetic genes for potential transcription factor binding sites. This analysis revealed multiple putative Vdr binding sites within the* ODC1*, the rate-limiting enzyme in SPD biosynthesis, promoter (Figure S3E). To investigate the binding of Vdr to the *ODC1* promoter, we designed a series of human *ODC1* promoter primers (Figure 3J, upper). We then performed chromatin immunoprecipitation (ChIP) in 293T cells transfected with either a Vdr overexpression plasmid or an empty vector control, using an anti-Vdr antibody to enrich bound chromatin. The ChIP results demonstrated significant enrichment of Vdr binding to the *ODC1* promoter region between -761 and -676 bp (amplified by primer F7) compared to the control (Figure 3J, lower). We transfected 293T cells with increasing concentrations of a Vdr overexpression plasmid (using Lipo2000: plasmid ratios of 1μL:0.75 μg, 1 μL:1 μg, and 1 μL:1.25 μg) and observed a corresponding upregulation of ODC1 protein levels (Figure 3K). To determine if this regulation occurred at the transcriptional level, we performed a bioinformatic analysis using JASPAR, which identified a putative Vdr binding sequence within the promoter region of the *ODC1* gene (specifically, the region amplified by primer F7) (Figure 3L). To test the function of the predicted Vdr-binding site, we constructed luciferase reporter plasmids carrying either the wild-type or a mutated version of the Vdr-like sequence (-761 to -754 bp) in the human *ODC1* promoter (Figure 3M). Luciferase assays confirmed the enhancer function of this element, as evidenced by significantly higher luciferase activity in 293T cells transfected with the wild-type plasmid compared to the control (pGL4.1-basic) (Figure 3N). In contrast, luciferase activity was significantly reduced in 293T cells transfected with a pGL4.1 plasmid containing mutated Vdr binding sites compared to those transfected with a plasmid carrying the intact Vdr binding sequence (Figure 3N), demonstrating that Vdr regulates ODC1 expression at the transcriptional level. In summary, our findings demonstrate that loss of *Vdr* alters the metabolic profile of granulosa cells and that Vdr transcriptionally regulates ODC1 expression, thereby modulating cellular SPD levels.

### SPD rescues abnormal ovarian development resulting from *Vdr* loss in GCs

**Figure 4:**
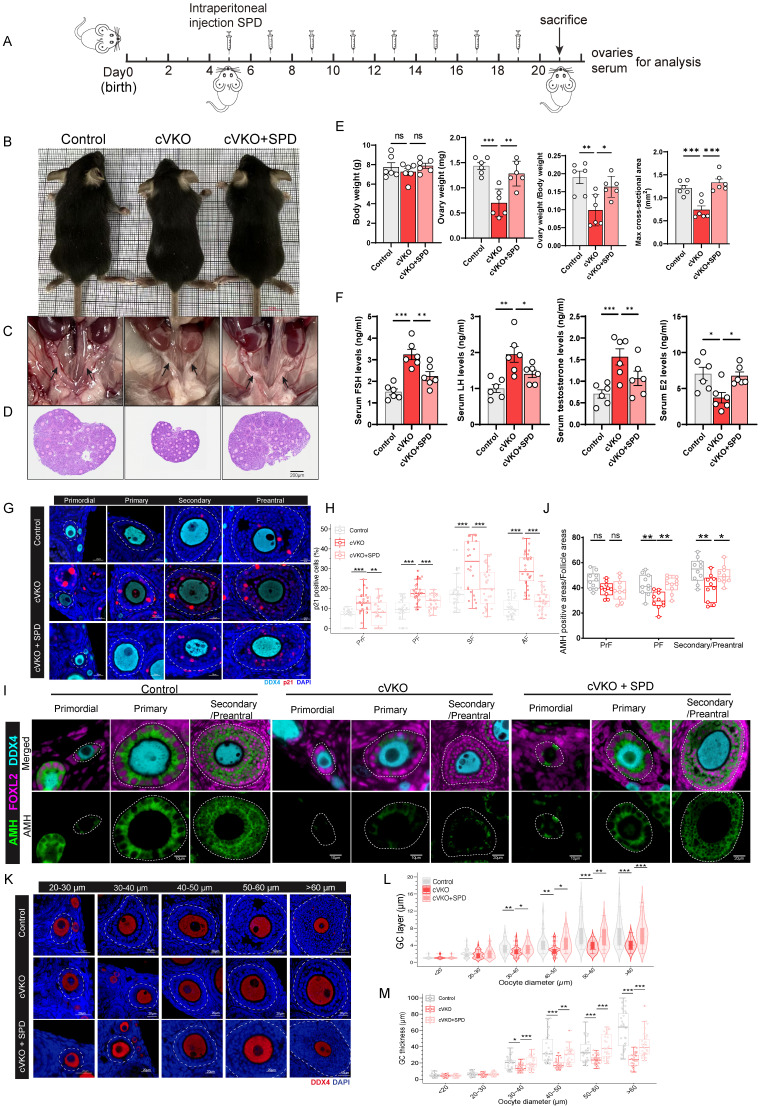
** The follicular developmental retardation induced by *Vdr* loss is linked to SPD 's anti-aging effect.** (A) Schematic of SPD injection workflow. (B) Representative appearance of 3-week-old control, cVKO, and cVKO mice treated with SPD. (C) Representative appearance of the uterus. (D) Representative micrographs of ovarian sections from the indicated group. (E) Body weight, ovary weight, ovary weight/body weight ratio, and max cross-sectional area of ovaries (n=6). (F) FSH, LH, E2, and testosterone levels in mice serum (n=6). (G) mIHC showing ovary staining for p21 and DDX4 at different developmental stages, with nuclei staining for DAPI. (H) Analysis for p21 positive cells (n=30). (I) mIHC showing ovary staining for Amh, Foxl2, and DDX4. (J) Analysis for Amh positive area relative to Follicle area (n=10). (K) mIHC showing ovary staining for DDX4 with different diameters of oocytes. (L) GC layer thickness categorized by oocyte diameter follicles (n=30). (M) GC thickness categorized by oocyte diameter follicles (n=30). Statistical analysis was performed with one-way ANOVA. Values are means ± SEM. *P < 0.05, **P<0.01, ***P < 0.001.

To further investigate whether *Vdr* loss in GCs leads to reduced ovarian SPD levels and impaired ovarian development, and whether SPD supplementation can mitigate GCs aging induced by loss of *Vdr*, we generated and genotyped mice with GC-specific knockout of *Vdr* (cVKO) and administered cVKO with SPD (Figure 1H and Figure 4A). The 3-week-old cVKO mice exhibited no significant difference in body size (Figure 4B and 4E), but showed reduced size of the uterus and ovaries (Figure 4C-4E), as well as a decreased ovarian index (ovary weight/body weight) (Figure 4E). The enzyme-linked immunosorbent assay was used to determine the levels of SPD in ovaries. The results showed that a substantial decrease in SPD level was observed in ovaries from cVKO mice compared to those from controls (Figure S4A). Obviously, intraperitoneal supplementation of SPD every 48 hours significantly elevated ovarian SPD levels in cVKO mice (Figure 4A and Figure S4A) and increased the size of the uterus and ovaries compared with controls (Figure 4C-4E and Figure S4F). We therefore proceeded to analyze key serum hormones derived from or associated with granulosa cells (GCs)-specifically estradiol (E2) and testosterone as well as gonadotropins [follicle-stimulating hormone (FSH) and luteinizing hormone (LH)], to gain further insight into GCs function in cVKO mice. The results demonstrated that cVKO mice exhibited elevated serum levels of FSH, LH and testosterone, accompanied by a reduction in E2 (Figure 4F). These hormonal imbalances were partially ameliorated following SPD supplementation, indicating a potential regulatory role of SPD in GCs function (Figure 4F). Since loss of *Vdr* was shown to promote GCs aging *in vitro* (Figure 1J and 1K), we next asked whether cellular aging is also increased in cVKO GCs *in vivo*. To address this, we examined the expression of the aging marker p21. We observed accelerated aging and reduced proliferative capacity of GCs in cVKO mice at multiple stages of folliculogenesis, as evidenced by increased p21 expression and a decreased number of Ki67-positive cells.

In contrast, SPD supplementation attenuated this aging phenotype and promoted the proliferative capacity of GCs (Figure 4G-4H, S4B-S4C and S4E). Given that GCs in cVKO mice exhibit signs of aging, we next sought to evaluate whether these cells maintain functional maturity. Anti-Müllerian hormone (Amh) is not initially expressed in pre-granulosa cells (pre-GCs) of primordial follicles; its expression initiates upon GCs growth and their transition to a cuboidal morphology^38^, ^39^. To this end, we examined the expression of Amh, a well-established functional marker of GCs^38^, ^40^. Foxl2 was also examined as a marker for pre-GCs/GCs in this analysis^38^. mIHC staining and western blot revealed reduced Amh expression in cVKO mice (Figure 4I-4J and Figure S4D-S4E). SPD supplementation was found to elevate Amh levels in GCs from primary to preantral follicles (Figure 4I-4J and Figure S4D-SE). Moreover, later stages of folliculogenesis are influenced markedly by GCs-derived paracrine factors and gonadotropins^41^. To investigate whether folliculogenesis defects are caused by GCs aging induced by *Vdr* loss, preantral follicles were classified into groups according to oocyte diameter: 20-30 μm, 30-40 μm, 40-50 μm, 50-60 μm and > 60 μm. Developmental differences in GCs between control and cVKO mice were then compared across these groups. The GCs morphology in these follicles was cuboidal or columnar, suggesting that the activation from primordial follicles (PrFs) to primary follicles (PFs), secondary follicles (SFs) and antral follicles (Afs) was normal in cVKO mice (Figure 4K). We quantified both GCs thickness and the number of GCs layers. A significant reduction in both the number of follicular GCs layers and GCs thickness was observed in the cVKO groups when the oocyte diameter exceeded 30μm (Figure 4L, 4M). As expected, these structural impairments resulting from *Vdr* loss were effectively restored following SPD supplementation (Figure 4K-4M). In conclusion, our findings demonstrate that the loss of *Vdr* promotes cellular aging and suppresses the proliferation of granulosa cells (GCs) in cVKO mice. These impairments in GCs function consequently hinder follicular development. Intraperitoneal administration of SPD effectively mitigates *Vdr* loss-induced GCs aging, enhances functional recovery of GCs, and thereby promotes follicular development.

### SPD attenuates GCs aging by promoting DNA methylation of p53 via DNA methyltransferases (DNMTs)

**Figure 5:**
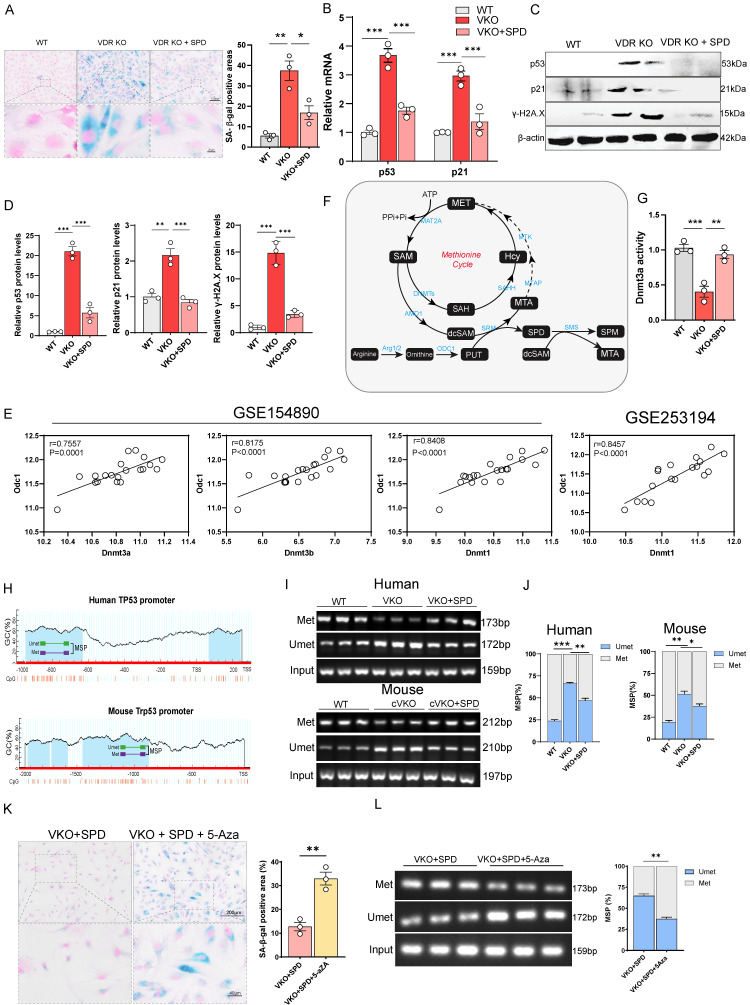
** SPD regulates GCs aging by modulating the DNA methylation through DNMTs.** (A) SA-β-gal staining for KGN cells, and analysis for SA-β-gal positive areas (n = 3). (B) *p53* and *p21* mRNA levels by real-time RT-PCR relative to *β-actin* mRNA in KGN cells (n = 3). (C-D) Western blots of KGN cell proteins showing p53, p21, and γH2A.X expression. Densitometric analysis was used to assess protein levels relative to β-actin (n = 3). (E) Scatter plot showing correlation between *ODC1* and *DNMTs*. (F) Schematic presentation of the methionine cycle. (G) Analysis of DNMT3A activity in KGN cells (n = 3). (H) Schematic representation of the mouse and human *TP53/Trp53* promoters. (I) Representative agarose gel analyses of MSP products from KGN cells or mice GCs. (J) Statistical analysis of MSP products (n = 3). (K) SA-β-gal staining for KGN cells, and analysis for SA-β-gal positive areas (n = 3). (L) Representative agarose gel analyses of MSP products from KGN cells and statistical analysis of MSP products. Statistical analysis was performed with one-way ANOVA. Values are means ± SEM. *P < 0.05, **P < 0.01, ***P < 0.001.

SPD, a well-documented anti-aging metabolite, is known to exert its anti-aging effects primarily through the induction of autophagy^24^, ^42^. Results from SA-β-gal staining, quantitative PCR, and immunoblotting showed that SPD supplementation attenuated aging in VKO cells by reversing the upregulation of p53 and p21 at both the transcriptional and protein levels in VKO cells (Figure 5A-5D). These findings suggest that SPD modulates the levels of aging-related proteins by regulating gene transcription through mechanisms independent of autophagy. To investigate the regulatory relationship between SPD and p53/p21, we investigated the association between epigenetic regulators and SPD. Correlation analysis of ovarian RNA-seq data indicated that ODC1 exhibited a strong correlation with DNA methyltransferases (DNMTs) (Figure 5E). Moreover, growth differentiation factor 9 (GDF9) and DDX4, which are established oocyte markers^38^, ^43^, ^44^, also showed strong correlations with DNMTs (Figure S5A), suggesting a potential functional role for DNMTs in follicular development. Elisa and immunoprecipitation results indicate that both the activity and protein levels of DNMTs were significantly reduced in VKO cells, and were restored by SPD supplementation (Figure 5G and S5B). This is consistent with previous studies suggesting that inhibition of ODC1 activity leads to decreased DNMTs activity^26^, ^45^.

Therefore, we hypothesize that the inhibition of SPD synthesis downregulates DNMTs activity, leading to reduced DNA methylation at aging-related gene loci such as p53 and p21. This hypomethylation is proposed to initiate their transcriptional activation, while SPD supplementation restores DNMTs activity, thereby suppressing their transcription. Using the MethPrime methylation prediction website, we identified a large CpG island within the promoter region of TP53/Trp53 (Figure 5H).

Methylation-specific PCR (MSP) analysis showed decreased methylation levels in the TP53/Trp53 promoter in both VKO and cVKO cells (Figure 5I and 5J). Thus, during *Vdr* loss-induced aging, the upregulation of p53 is likely attributable to reduced DNMTs activity, which results in hypomethylation of the p53 promoter. Additionally, increased methylation was observed in *Vdr*-loss cells following SPD supplementation, providing direct evidence that SPD influences *p53* promoter methylation (Figure 5I and 5J). Bisulfite sequencing PCR (BSP) analysis further confirmed a reduction in specific methylation within the p53 promoter, which could be restored by SPD supplementation (Figure S5C).

To further determine whether SPD alleviates aging induced by *Vdr* loss through the regulation of DNMTs, we treated the cells with 5-Azacytidine (5-Aza), a cytidine analog and known as a DNMTs inhibitor^46^. We aimed to determine whether DNMTs inhibition attenuates the anti-aging effects of SPD. SA-β-gal staining showed that the anti-aging effect of SPD was blocked by 5-Aza treatment in VKO cells (Figure 5K and S5D). Furthermore, MSP analysis revealed reduced methylation levels in the TP53 promoter in VKO+SPD cells (Figure 5L).

SPD is synthesized through the pathway in which methyl donor SAM is first decarboxylated to form decarboxylated SAM (dcSAM). Subsequently, dcSAM donates its aminopropyl group to putrescine, resulting in the formation of SPD (Figure 5F). DNMTs regulate methylation of DNA in the presence of SAM, while dcSAM is a strong inhibitor of DNMTs; the activity of DNMTs is closely associated with the dcSAM to SAM ratio^26^, ^47^, ^48^. To investigate the mechanism by which SPD promotes DNMTs activity, we hypothesize that the downregulation of ODC1 inhibits SPD synthesis, resulting in the accumulation of dcSAM, an elevated dcSAM/SAM ratio, and consequent inhibition of DNMTs activity. Additional SPD supplementation blocks the conversion of SAM to dcSAM by inhibiting Amd1 levels. To test this, shAMD1 was employed to block the conversion of SAM to dcSAM. SA-β-gal staining showed that blocking the conversion of SAM to dcSAM by knocking down the AMD1(Adenosylmethionine decarboxylase 1) could alleviate aging of VKO cells (Figure 5F and S5E) and enhance DNMTs activity (Figure S5F and S5I). The methylation level of the p53 promoter region was elevated by MSP analysis (Figure S5G), accompanied by increased expression of both p53 and p21 at the protein and mRNA levels (Figure S5H and S5I). Quantitative PCR analysis showed that additional SPD supplementation inhibits Amd1 levels (Figure S5J), suggesting SPD supplementation could block the conversion of SAM to dcSAM.

In summary, our data demonstrate that SPD supplementation could alleviate the aging of VKO cells mainly via increased DNMTs activity. Blocking SPD synthesis leads to dcSAM accumulation and downregulation of DNMTs activity.

### Supplementation with the direct methyl donor SAM improved follicular development in mice with granulosa cell-specific *Vdr* knockout

**Figure 6:**
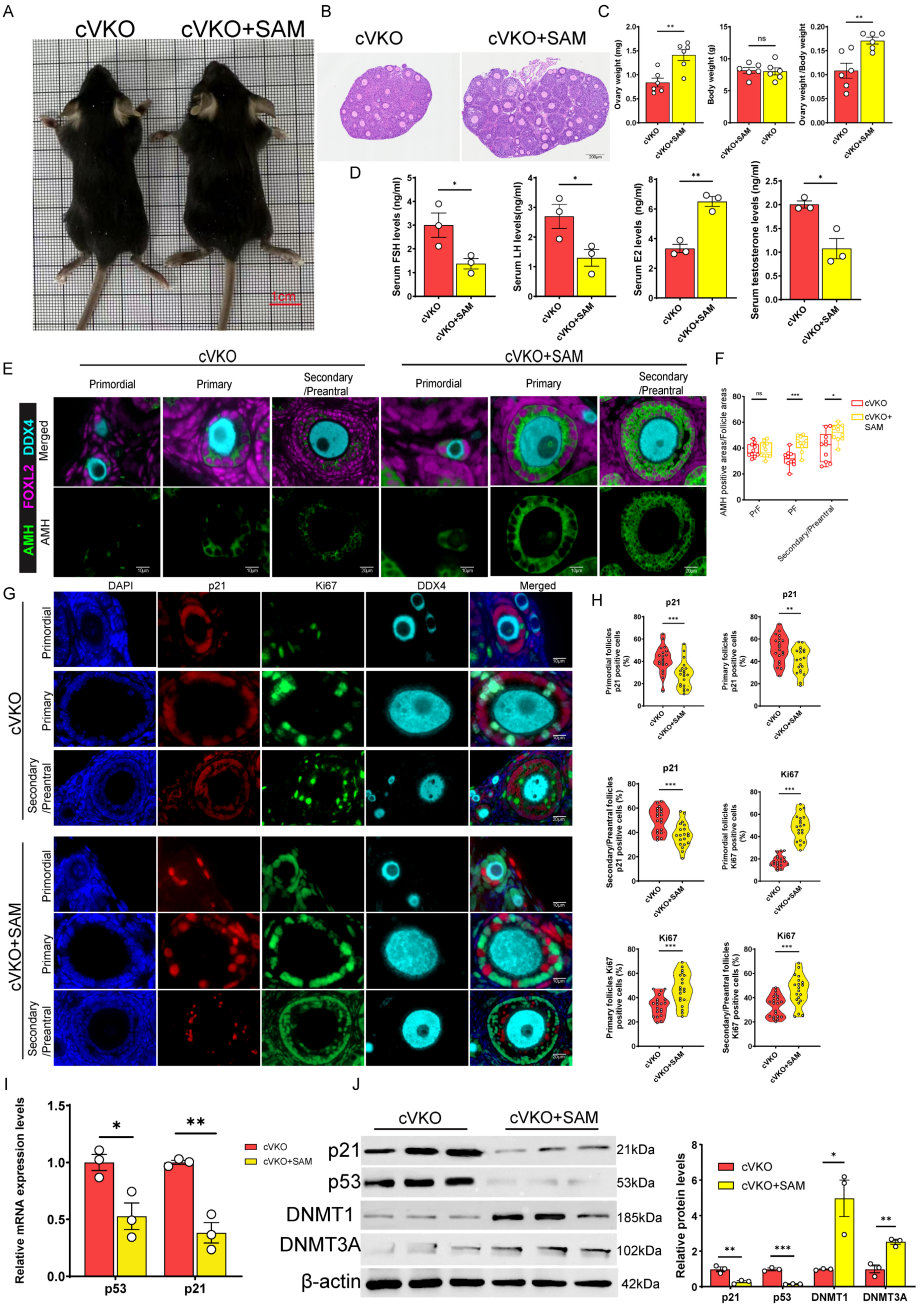
** SAM supplementation rescued ovary aging induced by *Vdr* loss in GCs.** (A) Representative appearance of 3-week-old cVKO and cVKO mice treated with SAM. (B) Representative micrographs of H&E staining for ovaries. (C) Ovary weight, body weight, and ovary weight/body weight ratio of mice (n = 6). (D) FSH, LH, E2, and testosterone levels in mice serum (n = 3). (E) mIHC showing ovary staining for Amh, Foxl2, and DDX4. (F) Analysis for Amh positive area relative to follicle area (n = 10) (G) mIHC showing ovary staining for p21, Ki67, and DDX4 at different developmental stages, with nuclei staining for DAPI. (H) Analysis for p21 and Ki67 positive cells (n = 20). (I) *p53* and *p21* mRNA levels by real-time RT-PCR relative to β-actin mRNA in ovaries (n = 3). (J) Western blots of ovary proteins showing p21, p53, DNMT1, and DNMT3A expression. Densitometric analysis was used to assess protein levels relative to β-actin. Statistical analysis was performed with an unpaired Student's t-test. Values are means ± SEM. *P < 0.05, **P < 0.01, ***P < 0.001.

Our findings indicate that DNMTs-mediated DNA methylation modulates p53 expression, which in turn directly influences follicular development. Additionally, the accumulation of dcSAM downregulates DNMTs activity, and this activity is strongly linked to the concentration of SAM^46^, ^49^. We assumed that SAM supplementation could restore DNMTs activity and thereby improve follicular development in cVKO mice. Our results demonstrated that SAM supplementation significantly increased the size and weight of the ovaries (Figure 6B and 6C), without affecting overall body size or body weight (Figure 6A and 6C). Hormone level tests result further indicated that cVKO mice treatment with SAM significantly reduced serum levels of FSH, LH, and testosterone, while increasing E2 levels (Figure 6D). mIHC staining and western blot showed that SAM supplementation promoted the Amh expression in cVKO ovaries (Figure 6E and S6A-S6B). We evaluated GCs status by staining for p21 and Ki67 proteins. Notably, SAM significantly increased the proportion of Ki67-positive cells and reduced the p21-positive cells in GCs from primordial to preantral follicular in cVKO mice (Figure 6G and 6H). Consistently, results from RT-qPCR and western blot showed that SAM supplementation downregulated the mRNA levels of *p53* and *p21*(Figure 6I) and p21 protein levels (Figure S6B). Immunoblotting results confirmed that SAM supplementation reduced the p53/p21 levels by affecting DNMTs levels (Figure 6J). In conclusion, our results suggest that SAM supplementation significantly ameliorates cVKO ovarian degradation by inhibiting GCs aging through DNMTs.

### Supplementation with SPD or SAM enhances DNMTs activity of aged ovaries, delays ovarian aging and promotes female fertility

**Figure 7:**
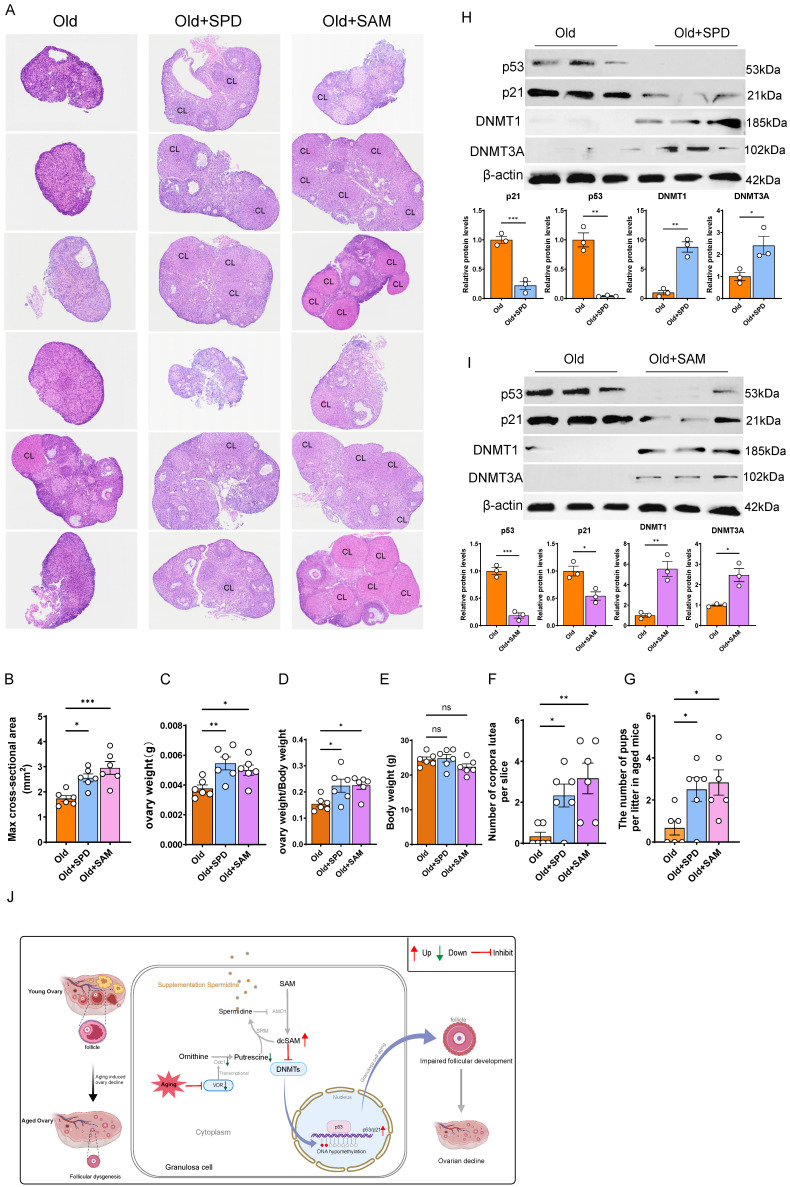
** SPD and SAM supplementation attenuate the ovarian aging process.** (A) Representative micrographs of H&E staining for ovaries in 12-month-old mice (Old) and 12-month-old mice treated with SPD or SAM, and CL indicating corpus luteum. (B) max cross-sectional area of the ovaries. (C-D) Analysis for ovary weight and ovary weight/body weight (n = 6). (E) Analysis for body weight. (F) Analysis for number of corpora lutea. (G) The litter size of the indicated female mice was quantified after mating with young male mice. (H-I) Western blots of ovary proteins showing p53, p21, DNMT1, and DNMT3A expression in old mice and old mice treated with SPD or SAM. Densitometric analysis was used to assess protein levels relative to β-actin (n = 3). (J) Schematic diagram of the mechanisms by which VDR regulates ovarian aging. Statistical analysis was performed with an unpaired Student's t-test for two group, one-way ANOVA for three group. Values are means ± SEM. *P < 0.05, **P < 0.01, ***P < 0.001.

Given that supplementation with SAM or SPD attenuated GCs aging and promoted follicular development in cVKO mice, we next investigated whether it exerted similar beneficial effects on the ovaries of aged mice. After 8-month-old female mice were supplement with SPD or SAM until to 12-month-old, ovaries were collected and the statistical data showed that SPD or SAM supplementation significantly increased the size and weight of the ovaries in aged mice (Figure 7A-7E and Figure S6F), without affecting overall body weight (Figure 7E).

Furthermore, while the ovarian tissue of aged mice exhibited a near-total loss of corpora lutea, this impairment was significantly rescued by supplementation with either compound, resulting in a substantial increase in corpora lutea number (Figure 7A and 7F). Accordingly, fertility tests revealed that the litter size of aged mice was considerably increased by supplementation with SPD or SAM (Figure 7G). To explore the potential mechanism, we performed immunoblotting to assess the effects of these supplements on DNMTs activity and p53/p21 levels. The results indicated that both SPD and SAM supplementation decreased p53/p21 levels and increased DNMTs levels in aged mice (Figure 7H and 7I). Furthermore, we examined whether supplementation with SPD or SAM could influence Vdr protein expression levels in aged ovarian tissues. The results showed that neither SPD nor SAM supplementation altered Vdr levels (Figure S6C). To further determine whether supplementation with SPD or SAM could ameliorate the decline in ovarian function caused by *Vdr* knockout from cVKO mice *in vitro*, we isolated ovarian tissues from 7-day-old female mice and cultured with SPD or SAM supplementation. Hematoxylin and eosin (H&E) staining and western blot analysis were performed. The results showed that SPD or SAM supplementation promoted ovarian growth in the cVKO group and significantly downregulated the expression levels of p53 and p21 in the ovaries of the cVKO group (Figure S6D and S6E). Collectively, these findings demonstrate that supplementation of SPD or SAM is a feasible strategy to delay ovarian aging and improve fecundity in aged female mice.

## Discussion

Ovarian aging is a key factor that impairs women's reproductive function. Emerging evidence indicates that epigenetic alterations, metabolic dysregulation, and gut microbial imbalance are among the most pivotal drivers of this aging process^18^, ^50^-^52^. The decline of ovarian function is driven by a reduction in both the quantity and quality of the ovarian follicle. Granulosa cells (GCs), as essential somatic components of follicles, play a critical role in follicular development and oocyte support^53^. Their functional integrity is paramount for female fertility. This study elucidates a novel Vdr-spermidine axis that orchestrates the maintenance of GCs homeostasis and protects against age-related ovarian decline through an epigenetic program mediated by DNA methyltransferases (DNMTs).

Our findings identify Vdr downregulation in GCs as a key driver of ovarian aging. We observed that Vdr is mainly expressed in ovarian GCs, consistent with previous reports^54^. Furthermore, Vdr expression gradually declines with age, and 1,25(OH)_2_D levels also decrease with age. This reduction coincides with increased levels of aging markers p53 and p21, suggesting a potential link between Vdr loss and GCs aging. To directly examine this hypothesis, we generated a granulosa cell-specific Vdr knockout (cVKO) mouse model and established a Vdr-knockout (VKO) human granulosa cell line. Both models provided evidence that Vdr deficiency accelerates GCs aging, as demonstrated by increased SA-β-gal activity, upregulation of p21, and decreased Ki67 expression. These findings establish Vdr as a pivotal anti-aging transcription factor in GCs. Given the recognized role of mitochondria in reproductive aging and the established role of Vdr in maintaining mitochondrial function through regulation of mitophagy^55^, ^56^, we found that Vdr loss induces profound mitochondrial dysfunction in GCs, including impaired oxidative phosphorylation, reduced mitochondrial biogenesis, and diminished mitochondrial quantity and quality. This aligns with the growing recognition of mitochondria as key players in reproductive aging^57^. These alterations disrupt cellular metabolic homeostasis and reinforce the role of mitochondrial dysfunction in GCs aging. To further elucidate the metabolic consequences of Vdr deficiency, we performed transcriptomic and metabolomic analyses. The results revealed extensive metabolic reprogramming, with polyamine metabolism emerging as a significantly disrupted pathway. Specifically, genes and metabolites involved in *de novo* SPD synthesis were coordinately downregulated, including the rate-limiting enzyme ornithine decarboxylase (ODC1). Correspondingly, intracellular SPD levels were markedly reduced in VKO cells and in the ovaries of cVKO mice. Mechanistically, we demonstrated that Vdr directly regulates ODC1 transcription. Bioinformatic prediction, chromatin immunoprecipitation (ChIP), and luciferase reporter assays confirmed that Vdr acts as a transcriptional activator of the ODC1 gene. These findings establish a direct molecular link between Vdr signaling and polyamine biosynthesis, revealing a previously unrecognized metabolic function of Vdr in the regulation of cellular aging. *In vivo* supplementation with SPD effectively ameliorated ovarian pathologies in cVKO mice, restoring ovarian and uterine size, improving hormonal profiles (decreased FSH, LH, and testosterone; increased E2), enhancing expression of the GCs functional marker Amh, and promoting follicular development through increased GCs proliferation. Importantly, SPD administration significantly attenuated GCs aging. Together, these findings support a model in which age-associated decline in Vdr expression leads to reduced SPD biosynthesis via transcriptional repression of ODC1, thereby driving GCs aging and ovarian functional deterioration. This model is consistent with previous studies showing that SPD supplementation promotes follicular development in aged female mice and that ODC1 overexpression enhances GCs proliferation^18^, ^58^, ^59^.

We then delved into the mechanistic pathway downstream of SPD. SPD has been reported to improve ovarian function through multiple mechanisms. Previous study has demonstrated that supplementation with SPD *in vivo* rejuvenated the quality of oocytes from aged mice by promoting autophagy, thereby increasing animal fertility^18^, ^60^. SPD supplementation improved ovarian endocrine function and reproductive capacity and alleviated oxidative stress by activating the Nrf2/HO-1/GPX4 pathway^32^. However, our data suggest that its anti-aging effects in this context are primarily mediated through an epigenetic pathway involving DNMTs. Correlation analysis of ovarian RNA-seq data indicated a strong positive association between *ODC1* and *DNMTs*. We found that *Vdr* knockout led to a significant decrease in both the activity and protein levels of DNMTs, which was reversible upon SPD supplementation. The fact that the DNMTs inhibitor 5-Azacytidine blocked the anti-aging effect of SPD further solidifies the necessity of DNMTs activity in this pathway. This aligns with existing literature suggesting a crosstalk between polyamine metabolism and DNA methylation^26^, ^47^. However, the role of SPD in promoting autophagy in GCs still needs further study.

The core of our proposed mechanism lies in the metabolic interplay between SPD synthesis and the methyl donor SAM. SPD biosynthesis consumes decarboxylated SAM (dcSAM), a potent inhibitor of DNMTs. This consumption thereby reduces the dcSAM/SAM ratio, whereas an increased ratio is known to inhibit DNMTs activity^25^, ^26^. We hypothesize that *Vdr* loss-induced suppression of ODC1 leads to an accumulation of dcSAM, thereby promoting the dcSAM/SAM ratio to inhibit DNMTs activity. The results from our *AMD1* knockdown experiment confirm this hypothesis, as reducing the conversion of SAM to dcSAM successfully ameliorated aging and enhanced DNMTs activity in VKO cells. SAM is not only an aminopropyl donor for polyamine synthesis, but it is also a methyl group donor for transmethylation and promotes DNMTs expression^46^, ^61^, ^62^. SAM supplementation counteracts the age-related decline in muscle regenerative capacity^63^ and mitigates the cachexia-induced atrophy by enhancing DNMT3A expression^46^, ^64^. Previous research has identified that reduced DNMTs expression during ovarian aging contributes to the development of female infertility^65^. We demonstrated that supplementation with SAM improved ovarian morphology, hormone levels, and reduced p53/p21 expression in ovaries from cVKO mice by increasing DNMTs expression. Most importantly, the translational potential of targeting this pathway was confirmed in naturally aged mice. Both SPD and SAM supplementation effectively delayed ovarian function decline in naturally aging mice, as indicated by increased ovarian weight, restoration of corpora lutea, and, most significantly, improved litter size in aged mice. The associated increase in DNMTs levels and decrease in p53/p21 levels in aged ovaries suggest a conserved mechanism across different models of ovarian aging in naturally aging mice and in cVKO mice.

In conclusion, our study identifies a novel Vdr-spermidine-DNMTs signaling axis that is essential for preserving GCs function and maintaining ovarian health (Figure 7J). We propose that age-related decline in Vdr expression initiates a cascade of events: reduced transcriptional activation of *ODC1*, diminished SPD production, accumulation of the DNMTs inhibitor dcSAM, inhibition of DNMTs activity, hypomethylation of the p53 promoter, and ultimately, induction of GCs aging and ovarian aging. Our work is the first to identify SPD as an accelerator of DNMTs and to propose both SPD and SAM as promising therapeutic candidates for extending the reproductive lifespan in women.

### Limitations of the study

Due to the current absence of direct quantitative methods for dcSAM, our indirect approach of knocking down its upstream synthesis genes, while informative, must be interpreted with caution. Finally, it is important to note that our study primarily relied on mouse models. Therefore, the conservation of the key molecular mechanisms we identified requires direct validation in humans.

## Materials and Methods

### Bioinformatics analysis

Identification of motifs was performed with the HOMER software suite. (http://homer.ucsd.edu/homer/microarray/index.html). We utilized R packages for bioinformatic analysis as follows: the Deseq2 package was employed to identify differentially expressed genes (DEGs) under the criteria of |log2foldchange|≥ 1 and adjusted p-value (p.adj) < 0.05. Subsequently, the ClusterProfiler package facilitated Gene Ontology (GO) analysis and Gene Set Enrichment Analysis (GSEA), while the ggplot2 package was used for data visualization.

### *VDR* knock-out cell generation

*VDR* knock-out KGN cells were carried out by the epiCRISPR system as previously reported^66^. Single-guide RNAs (sgRNAs) targeting human *VDR* were subcloned into the epiCRISPR vector digested by BspQI (NEB, R0712S). The two plasmids were co-transfected into KGN cells for 48 hours, and then puromycin (0.5μg/ml) was administered to screen for positive cells for 1 week. The single cells were then isolated in 96-well plates. KO cells were validated by agarose gel and WB analysis. The sequences of the sgRNA are listed as follows. sgVDR1 F 5'-caaagtctccagggtcaggc-3' R 5'-gcctgaccctggagactttg-3' and sgVDR2 F 5'-tcacaggtcatagcattgaa-3' R 5'-ttcaatgctatgacctgtga-3'.

### Mice and genotyping

The *Cyp19a1Cre* mice (C57BL/6JCya-Cyp19a1*^tm1(IRES-CRE)^*/Cya, NO. C001382) and *Vdr-flox* mice (C57BL/6JCya-*Vdr^em1flox^*/Cya, Strain NO. S-CKO-17422) used in this study were purchased from Cyagen. All mice were maintained under specific pathogen-free (SPF) conditions with a 12/12-hour light/dark cycle at 21-24 °C and had ad libitum access to standard rodent chow and water. For euthanasia, mice were administered an overdose of isoflurane. All experimental procedures were conducted in accordance with humane ethical guidelines. Animal experiments were performed following protocols approved by the Institutional Animal Care and Use Committee of Nanjing Drum Tower Hospital, Nanjing University Medical School (2024AE01082).

The *Cyp19a1Cre* allele was detected using the *Cyp19a1Cre* forward 5´-CATATTGGCAGAACGAAAACGC-3´and reverse 5´- CCTGTTTCACTATCCAGGTTACGG-3´ primers. The *loxp1* allele of VDR heterozygous (VDR f/+) mouse was detected using the forward 5´-TAAAGCTTCACCACCCCTAATAGC-3´ and reverse 5´- GAAGTACCTTTTACTGGCTTTGGAC-3´ primers. The *loxp2* allele was detected using the forward 5´-GATGTTTCATGGGAGGAATAAGCAG-3´ and reverse 5´- CAGTGACTAGGTAGCAGCAATGAC-3´ primers. The VDR f/f mice (Control) used in the experiments were obtained from mating male and female VDR f/+ mice. The VDR f/f mice were crossed with *Cyp19a1Cre* mice and mated again to generate VDR f/f Cyp19a1Cre^+^ mice (cVKO).

### Animal experiments

To investigate the effects of SPD and SAM on the ovaries, we supplemented aged mice and cVKO mice with SPD and SAM respectively, with specific measures as follows: Aged female mice were supplemented with SPD (3 mM, Psaitong, S50085) from 8-month-old in drinking water as previously reported for 4 months^18^. Aged female mice received intraperitoneal administration of the SAM (10 mg/kg) three times per week. Due to the inability of neonatal mice to freely access food and water, we administered the treatment via intraperitoneal injection. Control and cVKO female mice received intraperitoneal injections of either SAM (Selleck, S5109) at 10 mg/kg or SPD (Psaitong, S50085) at 50 mg/kg^18^, ^46^ every other day.

To assess fertility, aged female mice were continuously housed with young wild-type males, and the resulting pups were counted.

### Measurement of relative mtDNA content

To evaluate mitochondrial quantity, the relative mitochondrial DNA (mtDNA) copy number was quantified by quantitative PCR. Briefly, total DNA was extracted from cells with a commercial DNA extraction kit (Beyotime). Using 10 ng of the DNA, mitochondrially encoded genes (MT-CO1, MT-ND1, MT-CO3, and the D-loop region) were amplified and normalized to the nuclear reference gene β2-microglobulin (β2-MG). The corresponding primer sequences are listed in Supplementary Table S2.

### SA-β-gal staining

We employed SA-β-gal staining to evaluate and quantify cellular senescence, with the specific protocol as follows: For freshly cultured cells, aspirate the culture medium, wash three times with PBS, add 1 mL of β-galactosidase staining fixative solution, and fix at room temperature for 20 minutes. Aspirate the cell fixative solution, and wash the cells three times with PBS, each time for 3 minutes. Aspirate the PBS, and add 0.5 mL of the staining working solution to each well. Incubate at 37 °C overnight, sealing the plate with Parafilm or plastic wrap to prevent evaporation, then observe and photograph under a microscope. The SA-β-gal staining was conducted using the kit (Beyotime, C0602).

### RNA-Sequencing

Total RNA extraction, library construction, and sequencing were performed by Novogene Co., Ltd. (Beijing, China). Briefly, total RNA was isolated with Trizol reagent and assessed for quality. Eukaryotic mRNA was then enriched with Oligo (dT) beads and fragmented. Using the NEBNext Ultra RNA Library Prep Kit for Illumina (NEB #7530), the fragmented mRNA was precipitated with an equal volume of isopropanol and subsequently underwent phosphorylation and polyadenylation. The cDNA library was generated by RT-PCR, and then amplified by PCR to ligate the adaptor. The PCR product was purified and retracted by an 8% TBE gel. Finally, the prepared library was sequenced on an Illumina NovaSeq 6000 platform.

### Targeted metabolomics

The procedures for metabolite extraction and data analysis were outsourced to Personalbio (Nanjing, China). The extraction protocol comprised the following steps: First, A total of 1 × 10^7 cells per sample were collected and snap-frozen in liquid nitrogen. Next, cells were aliquoted on dry ice into a 2 mL tube. Subsequently, 1000 μL of cold extraction solvent (methanol/acetonitrile/water, 2:2:1, v/v), pre-mixed with stable-isotope internal standards for quantification, was added and thoroughly vortexed. The samples then underwent homogenization, followed by sonication at 4 °C and centrifugation. The clarified supernatant was dried in a vacuum centrifuge. Finally, for LC-MS analysis, the samples were reconstituted in 100 μL of acetonitrile/water (1:1, v/v), centrifuged, and the supernatant was injected. Metabolomic data were processed using MultiQuant or Analyst software. Quality control (QC) samples were analyzed alongside the biological samples, and metabolites with a coefficient of variation (CV) < 30% in the QCs were considered reproducible. The filtered dataset was first subjected to principal component analysis (PCA) to assess sample grouping, followed by orthogonal projections to latent structures-discriminant analysis (OPLS-DA) to obtain variable importance in projection (VIP) scores. Differential metabolites were subsequently identified by combining VIP scores from the OPLS-DA model with univariate statistical analysis. The results of targeted metabolomics are shown in Supplementary Table S1. The result of enrichment using differential metabolites are shown in Supplementary Table S3.

### Isolation of primary granulosa cells (GCs)

Two-month-old female mice received a 20 IU injection of pregnant mare serum gonadotropin (PMSG) to stimulate multi-follicular development. Forty-eight hours thereafter, ovaries were collected for granulosa cell (GCs) isolation. The cell suspension was filtered through a 70-μm cell strainer to remove debris. Primary GCs were then cultured in DMEM-F12 medium supplemented with 10% fetal bovine serum (FBS, SA211.02, CellMax, USA) and 1% penicillin-streptomycin (15140-122, Gibco, USA), and maintained at 37 °C in a 5% CO₂ atmosphere.

### Cell culture and treatment

The human granulosa-like tumor cell line KGN and the human embryonic kidney HEK-293T cells were maintained at 37 °C in a 5% CO₂ atmosphere. KGN cells were cultured in DMEM/F12 medium, while HEK-293T cells were cultured in DMEM. Both media were supplemented with 10% fetal bovine serum (FBS) and 1% penicillin-streptomycin. Spermidine (20 μM, Psaitong, S50085), 5-Aza (5 μM, MCE, HY-10586) or SAM (100 μM, Selleck, S5109) were added into GCs or KGN cells.

### Methylation-specific PCR (MSP) and bisulfite sequencing PCR (BSP)

Methylation-specific PCR (MSP) and bisulfite sequencing PCR (BSP) assays were performed to analyze promoter methylation. Primers for human and mouse p53 promoters were designed following CpG island prediction by the online MetPrimer program (http://www.urogene.org/methprimer). MSP amplification products were electrophoresed on 2% agarose gels, and band intensities were quantified using ImageJ software after UV visualization. For BSP, bisulfite conversion of genomic DNA was carried out with a commercial kit (Vazyme, EM102-01). The converted DNA was amplified, and the PCR products were purified, cloned into a sequencing vector, and subjected to sequencing. Methylation was calculated as the percentage of methylated cytosines across all CpG sites, with results depicted graphically in a figure where each row of dots represents a single clone. Corresponding primers can be found in Supplementary Table S2.

### *AMD1* knockdown cell generation

For knockdown of AMD1 in KGN cells, lentiviruses expressing shRNAs targeting human AMD1 were generated. Two shRNA sequences (5'-TCTGACTGTTGGTACTTATAT-3' and 5'-GATGGAACTTATTGGACTATT-3') were synthesized (Sagon Biotech, Shanghai, China), annealed, and then ligated into AgeI and EcoRI sites of pKLO.1. shRNA expression plasmid. Lentiviral particles were produced by co-transfection of pLP1, pLP2, VSV-G, and pKLO.1-shRNA plasmids at a 1:1:1:3 mass ratio into HEK293T cells. Supernatants were collected 48 h post-transfection, filtered through 0.45 µm sterile SFCA filters, and either used immediately or stored at -80 °C. For stable cell line generation, lentiviral supernatants were mixed with fresh medium at a 1:1 ratio and applied to cells. After 48 h of infection, the media were replaced, and stable clones were selected with 0.8 µg/mL puromycin for at least 4 days. Knockdown efficiency was confirmed by immunoblotting.

### ChIP-qPCR

Chromatin immunoprecipitation (ChIP) was performed on KGN cells using the Magna ChIP™ kit (Millipore). Sheared chromatin was immunoprecipitated with an anti-Vdr antibody (ab3508, Abcam) or a normal rabbit IgG (#PP64, Millipore) as a negative control. Precipitated DNA was analyzed by qPCR with primers targeting different regions of the VDR promoter (see Supplementary Table S2).

### Dual-Luciferase assay

For the luciferase reporter assay, HEK-293T cells were seeded in 24-well plates and co-transfected with either the ODC1-pGL4.1 promoter construct or its mutant (ODC1-mut-pGL4.1), along with the Rluc-pGL4.1 control plasmid. After 24 hours, cells were washed with PBS, and luciferase activity was measured using a dual-luciferase assay kit (Promega, E1960) according to the manufacturer's instructions. Luminescence signals were quantified with a BMG LABTECH microplate reader.

### RNA extraction and quantitative real-time PCR

Total RNA was extracted using TRIzol LS. For each sample, 1 μg RNA was reverse-transcribed using the HiScript III RT SuperMix (Vazyme, #R323-01). qPCR was conducted using ChamQ Universal SYBR qPCR Master Mix (Vazyme, #Q711-02). Gene expression was quantified using the 2^-ΔΔCt^ method with primers listed in Supplementary Table S2.

### Multiple-Colored Immunohistochemistry (mIHC)

Serial paraffin sections were deparaffinized and rehydrated for histochemical or immunofluorescence staining using the TSA kit (Panovue, #0079100020). In brief, paraffin sections were microwaved in Tris-EDTA (pH 9.0) to retrieve antigens. After blocking with 5% BSA (additionally with 0.04% Triton X-100 in PBS) for 60 minutes at room temperature, sections were incubated with the first primary antibody at 4℃ overnight. After washing with PBS, incubating the sections with HRP-conjugated secondary antibody for 1 h at room temperature and then with various PPD dyes (diluted in signal amplification solution) for 10 minutes. Repeating antigen retrieval, and incubating sections with the next antibody and PPD dyes until all the antibodies and dyes were incubated. After washing with PBS, incubating with DAPI for 10 minutes. Imaging was performed with an Olympus FV3000 confocal microscope. Specific antibodies against VDR (1:100, ab3508, Abcam), DDX4 (1:500, ab27591, Abcam), Ki67 (1:200, ab16667, Abcam), p21 (1:200, ab188224, Abcam), Amh (1:300, ab272221, Abcam), Foxl2 (1:400, ab246511, Abcam) were used.

### Histochemical and quantitative analysis of the follicle

Ovarian tissue sections were deparaffinized and rehydrated for histological analysis. For histological evaluation, sections were subjected to standard haematoxylin and eosin (H&E) staining. For quantitative follicle analysis, every fifth serial section was immunostained with an anti-DDX4 antibody (1:500; ab27591, Abcam) to mark germ cells. Only follicles containing an oocyte with a visible nucleolus were counted. Raw follicle counts were multiplied by five to estimate the total number per ovary. Follicles were classified into four developmental stages: primordial (an oocyte surrounded by a single layer of flattened pre-granulosa cells), primary (a single layer of cuboidal granulosa cells), secondary/preantral (two or more layers of granulosa cells without an antrum), and antral (multiple granulosa cell layers with a visible antral cavity). Granulosa cell thickness was measured in follicles with clear oocyte nuclei; quantitative analysis of follicle numbers and quantification of granulosa cell thickness are mainly based on established methods^37^, ^38^. Measurements were obtained using OlyVIA software, photographed with an Olympus VS200.

### Western blot analysis

Cells or tissues were lysed on ice for 15 minutes using RIPA buffer supplemented with protease and phosphatase inhibitors. The lysates were centrifuged at 13,000 rpm for 15 min at 4 °C, and the resulting supernatants were collected. After mixing with 5× SDS loading buffer and boiling at 100 °C for 10 min, the protein samples were separated by SDS-PAGE and transferred to PVDF membranes. The membranes were blocked with 5% skimmed milk in TBST for 1 hour at room temperature and then incubated with primary antibodies (diluted in 5% skimmed milk/TBST) overnight at 4 °C. Following three 10-minute washes with TBST, the membranes were incubated with HRP-conjugated secondary antibodies for 1 hour at room temperature. After washing, protein signals were captured with chemiluminescence. Specific antibodies against VDR (1:100, ab3508, Abcam), PGC1a (1:1000, 66369-1-Ig, Proteintech), AMPKa (1:1500, ab32047, Abcam), p-AMPKa (1:1500, ab133448, Abcam), ODC1(1:2000, ab270268, Abcam), Phospho-Histone H2A.X (1:2000, 9718T, CST), DNMT1 (1:1000, 5032T, CST), DNMT3A (1:1000, 3598T, CST), p21 (1:1000, 10355-1-AP, Proteintech), p21 (1:1000, ab188224, Abcam), p53 (1:1500, ab26, Abcam), Ki67 (1:500, ab16667, Abcam), Amh (1:1000, Proteintech, 84153-5-RR), Atp5a(1:1000, Proteintech, 14676-1-AP), Ndufb8 (1:1000, Proteintech, 14794-1-AP), Drp1 (1:1000, Proteintech, 12957-1-AP), mt-co1 (1:1000, CST, #62101), Mfn2 (1:1000, Proteintech, 12186-1-AP), Sdhb (1:1000, Proteintech, 10620-1-AP), Ass1 (1:1000, Proteintech, 16210-1-AP) Smox (1:1000, Proteintech, 15052-1-AP), Slc7a2 (1:1000, Proteintech, 30232-1-AP) β-actin (1:5000, 66009-1-Ig, Proteintech) were used.

### Measurement of hormone, SPD and ATP

To investigate the hormone levels in the serum samples, blood samples were centrifuged at 5000 rpm for 10 min to collect the serum, hormone levels were measured using the Cobas e602 module of the Cobas 8000 total automation system (Roche). To investigate the SPD levels in the ovarian samples, ovary tissue was homogenized in PBS; after lysis, centrifuge at 12,000g for 5 minutes at 4 ℃ and collect the supernatant for further quantification of SPD by an enzyme-linked immunosorbent assay kit (Cloud-Clone) according to the manufacturer's instructions. Cell ATP content was measured using an ATP assay kit according to the manufacturer's instructions (Beyotime, S0027).

### Oxygen consumption rate (OCR)

To investigate cellular mitochondrial function, we utilized the Seahorse analyzer to assess cellular respiration. Cellular oxygen consumption rate (OCR) was assessed with Agilent Seahorse XF Cell Mito Stress Test Kit (Agilent Technologies, 103015-100). In brief, 15,000 cells were seeded per well in a specialized microplate. On the assay day, the growth medium was replaced with pre-warmed Seahorse assay medium, and OCR was monitored using the XF Cell Mito Stress Kit. The assay protocol involved sequential injection of metabolic modulators: first, oligomycin (1.5 μM) was added to inhibit ATP synthase and reveal ATP-linked respiration; next, FCCP (1 μM) was introduced to uncouple mitochondria and measure maximal respiratory capacity; finally, a cocktail of antimycin A and rotenone (0.5 μM) was used to inhibit the electron transport chain, allowing quantification of non-mitochondrial oxygen.

### Flow cytometry

To evaluate mitochondrial quality and quantity of GCs, 100 nM mitotracker green (C1048, Beyotime) or mitotracker red (C1049, Beyotime) was used to incubate GCs for 15 minutes at 37℃. A positive signal was detected by FITC or PE with a FACSCalibur (BD) and analyzed with FlowJo software.

### Confocal microscopy

To evaluate mitochondrial quality and quantity of KGN cells, cells were seeded in a confocal dish, after being washed with PBS twice, stained with 100 nM mitotracker green and mitotracker red for 15 mins at 37 ℃. Hoechst 33342 was applied to stain for nuclei. Images were obtained with an Olympus FV3000 confocal microscope.

### Ovary culture *in vitro*

Intact ovaries were harvested from 7-day-old mice through micro-dissection in chilled phosphate-buffered saline (PBS). These isolated organs were subsequently maintained for 7 days on 0.4 µm pore-sized inserts (Millipore, Cat#PICM0RG50) within 6-well plates (NEST, Cat#703002). The primary incubation environment consisted of 3 mL DMEM/F12 containing 1% penicillin-streptomycin. Ovaries were subjected to treatment with either 20µM SPD or 100µM SAM. Half of the culture volume was replenished with fresh medium every 48 hours. Standard incubation conditions were set at 37 °C with a 5%CO_2_.

### Statistical analysis

All analyses were performed using GraphPad Prism software (Version 10; GraphPad Software Inc., San Diego, CA, USA). Measurement data are described as the mean ± SEM fold-change over the vehicle group were analyzed by using Student's t test and one-way ANOVA to compare differences among groups. Qualitative data are described as percentages and were analyzed using chi-square tests as indicated. P values were two-sided and a P value < 0.05 was considered statistically significant. Correlations of Gaussian-distributed data were analyzed by Pearson's *r*; non-Gaussian distributed data were analyzed by Spearman's rank correlation coefficient. P-values were two-sided and values less than 0.05 were considered statistically significant.

## Supplementary Material

Supplementary figures.

Supplementary tables.

## Data Availability

All additional datasets included in the manuscript will be provided upon request from the lead contact. The RNA-seq data generated in this study have been deposited in the National Genomics Data Center of China (NGDC) under the accession number PRJCA048546; the metabolome data were listed in Supplementary Table S1.
