# Supplementary material for: VDR-Spermidine Axis Protects Against Age-Related Granulosa Cell Dysfunction and Follicular Decline via DNMTs-Mediated p53 Methylation
Source: Int J Biol Sci. 2026 May 18;22(10):5399–420. doi: 10.7150/ijbs.128631 (PMC13215359; doi:10.7150/ijbs.128631)
Supplement: Supplementary file 1 — Supplementary figures. [file ijbsv22p5399s1.pdf]

Figure S1 Verification of *Vdr* knockout by genotyping in mice and KGN cells

(A) mRNA levels by real-time RT-PCR relative to *β-actin* mRNA in ovaries from young and aged mice (n=3). (B) Western blots of GCs proteins showing p53, p21, and Vdr expression in 2-month-old and 2-month-old with SPD or SAM treatment. Densitometric analysis was used to assess protein levels relative to β-actin (n=3). (C) Representative agarose gel analyses for cVKO mice genotype. (D) Schematic diagram of VKO KGN cells construction. (E) Representative agarose gel analyses for VKO KGN cells *Vdr* knockout identification (deletion length =111bp). (F) Western blots of VKO cell proteins showing Vdr expression. (G) Elisa for 25(OH)D_3 and 1,25$(OH)_2$D levels. (H) Western blots showing Vdr expression in KGN cells with or without 1,25$(OH)_2$D treatment. (I) Western blots of GCs proteins showing Amh, p21, and Ki67 expression in Control and cVKO mice. Statistical analysis was performed with an unpaired Student's t-test. Values are means ± SEM. *$P < 0.05$, **$P<0.01$, ***$P < 0.001$.

Figure S2 Aging downregulated the quantity and quality of mitochondria

(A) Heatmap showing changes of genes related to ETC and TCA in aged ovaries. (B) GSEA analysis showed abnormality of mitochondria in VKO cells. (C) Western blots of GCs proteins showing Atp5a, Ndufb8, Drp1, mt-co1, Mfn2 and Sdhb expression in Control and cVKO mice. Statistical analysis was performed with an unpaired Student's t-test. Values are means ± SEM. *$P < 0.05$, **$P<0.01$, ***$P < 0.001$.

Figure S3 Loss of *Vdr* disrupts polyamine metabolism

(A) Heatmap showing differential metabolites in VKO cells. (B) Principal-component (PC) analysis of transcriptome data from VKO and WT cells. Blue circles represent samples from the VKO group, and red circles represent samples from the WT group. (C) Volcano plot displays differential metabolites (upregulated, red; downregulated, blue) in VKO cells compared to those from WT. (D) Differential abundance score analysis of differential metabolites (upregulated, blue; downregulated, red) from VKO cells compared to those from WT ones. (E) Predicted Vdr binding site in the *Odc1* promoter region. (F) Western blots of GCs proteins showing Odc1, Ass1, Smox and Slc7a2 expression in Control and cVKO mice. Statistical analysis was performed with an unpaired Student's t-test. Values are means ± SEM. *P < 0.05, **P<0.01, ***P < 0.001.

Figure S4 SPD treatment rescued aging induced by *Vdr* loss in the ovaries

(A) SPD levels in the indicated ovaries. (B) Immunofluorescence showing ovary staining for DDX4 and Ki67. (C) SA-β-gal staining for primary ovarian granulosa cells. (D) Immunofluorescence showing ovary staining for Amh. (E) Western blots of GCs proteins showing Amh, p21, and Ki67 expression in Control and cVKO mice. (F) Gross photograph of ovarian tissue. Statistical analysis was performed with one-way ANOVA for three group. Values are means ± SEM. *P < 0.05, **P<0.01, ***P < 0.001.

Figure S5 SPD regulated GCs aging by modulating the DNA methylation through DNMTs

(A) Scatter plot showing correlation between *DNMTs* and *Gdf9* or *Ddx4* expression across transcriptomic datasets. (B) Western blots of KGN cell proteins showing p53, p21, and DNMTs expression. (C) Dot plots showing DNA methylation in KGN cells (n=5). Statistical analysis was performed with one-way ANOVA. Values are means ± SEM. ***P < 0.001. (D) Western blots of KGN cell proteins showing p21 and p53 expression. Densitometric analysis was used to assess protein levels relative to β-actin (n=3). (E) SA-β-gal staining for KGN cells, and analysis for SA-β-gal positive cells (n=3). Statistical analysis was performed with an unpaired Student's t-test. Values are means ± SEM. **P<0.01. (F) Analysis of DNMTs activity in KGN cells (n=3). (G) Representative agarose gel analyses of MSP products from KGN cells and statistical analysis of MSP products. Values are means ± SEM. **P<0.01. (H) *p53* and *p21* mRNA levels by real-time RT-PCR relative to β-actin mRNA in KGN cells (n=3). (I) Western blots of KGN cell proteins showing p21, p53, and DNMT3A expression. Densitometric analysis was used to assess protein levels relative to β-actin (n=3). (J) *Amd1* mRNA levels by real-time RT-PCR relative to β-actin mRNA in KGN cells (n=3). Statistical analysis was performed with an unpaired Student's t-test. Values are means ± SEM. *P < 0.05, **P<0.01, ***P < 0.001.

Figure S6 SPD or SAM supplementation rescue ovarian aging

(A) Immunofluorescence showing ovary staining for Amh. (B) Western blots of GCs proteins showing p21, and Amh expression. Densitometric analysis was used to assess protein levels relative to β-actin (n=3). (C) Western blot of ovarian proteins showing

Vdr expression. (D) H&E staining showing ovaries culture in vitro, and analysis of max cross-sectional area. (E) Western blots of GCs proteins showing p53 and p21 expression. Densitometric analysis was used to assess protein levels relative to β-actin (n=3). (F) Gross photograph of ovarian tissue. Statistical analysis was performed with one-way ANOVA for three group. Values are means ± SEM. $^{**}P<0.01$, $^{***}P < 0.001$.

S1

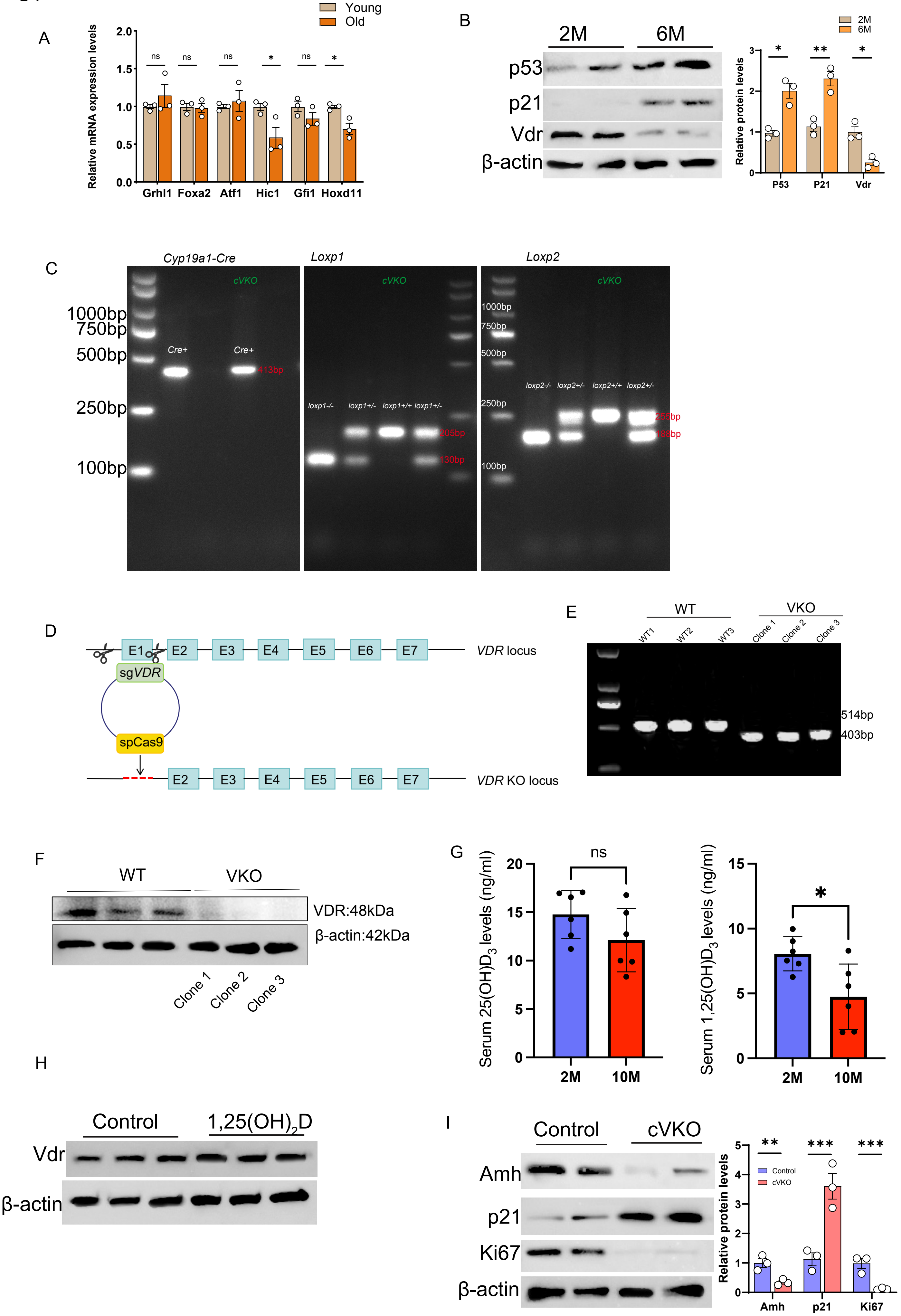

A
Young
Old
Relative mRNA expression levels
Grhl1 Foxa2 Atf1 Hic1 Gfi1 Hoxd11
ns ns ns * ns *
B
2M 6M
p53
p21
Vdr
β-actin
Relative protein levels
P53 P21 Vdr
C
Cyp19a1-Cre
Loxp1
Loxp2
cVKO
1000bp
750bp
500bp
250bp
100bp
Cre+
413bp
loxp1-/- loxp1+/- loxp1+/+ loxp1+/-
205bp
130bp
loxp2-/- loxp2+/- loxp2+/+ loxp2+/-
D
E1 E2 E3 E4 E5 E6 E7
VDR locus
sgVDR
spCas9
VDR KO locus
E
WT VKO
WT1 WT2 WT3 Clone 1 Clone 2 Clone 3
514bp
403bp
F
WT VKO
VDR:48kDa
β-actin:42kDa
Clone 1 Clone 2 Clone 3
G
Serum 25(OH)D3 levels (ng/ml)
Serum 1,25(OH)D3 levels (ng/ml)
2M 10M
ns
*
H
Control
1,25(OH)2D
Vdr
β-actin
I
Control cVKO
Amh
p21
Ki67
β-actin
Relative protein levels
Control
cVKO
Amh p21 Ki67
** *** ***

S2

A

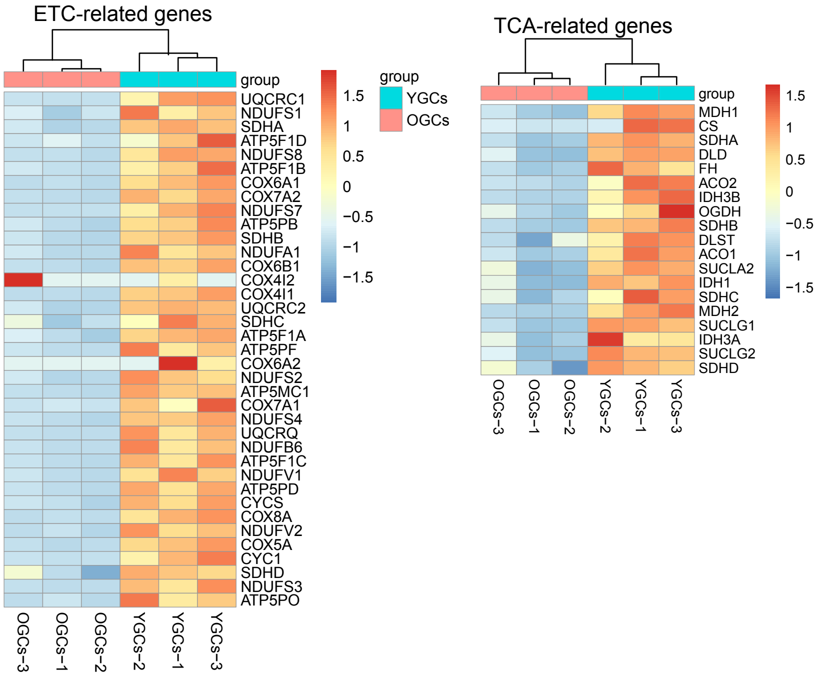
ETC-related genes
group
UQCRC1
NDUFS1
SDHA
ATP5F1D
NDUFS8
ATP5F1B
COX6A1
COX7A2
NDUFS7
ATP5PB
SDHB
NDUFA1
COX6B1
COX4I2
COX4I1
UQCRC2
SDHC
ATP5F1A
ATP5PF
COX6A2
NDUFS2
ATP5MC1
COX7A1
NDUFS4
UQCRQ
NDUFB6
ATP5F1C
NDUFV1
ATP5PD
CYCS
COX8A
NDUFV2
COX5A
CYC1
SDHD
NDUFS3
ATP5PO
OGCs−3
OGCs−1
OGCs−2
YGCs−2
YGCs−1
YGCs−3
1.5
1
0.5
0
−0.5
−1
−1.5
group
YGCs
OGCs
TCA-related genes
group
MDH1
CS
SDHA
DLD
FH
ACO2
IDH3B
OGDH
SDHB
DLST
ACO1
SUCLA2
IDH1
SDHC
MDH2
SUCLG1
IDH3A
SUCLG2
SDHD
OGCs−3
OGCs−1
OGCs−2
YGCs−2
YGCs−1
YGCs−3

B

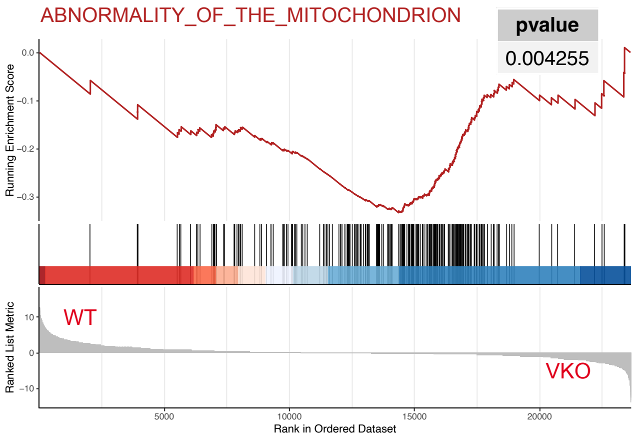
ABNORMALITY_OF_THE_MITOCHONDRION
pvalue
0.004255
Running Enrichment Score
0.0
−0.1
−0.2
−0.3
WT
VKO
Ranked List Metric
10
0
−10
5000
10000
15000
20000
Rank in Ordered Dataset

C

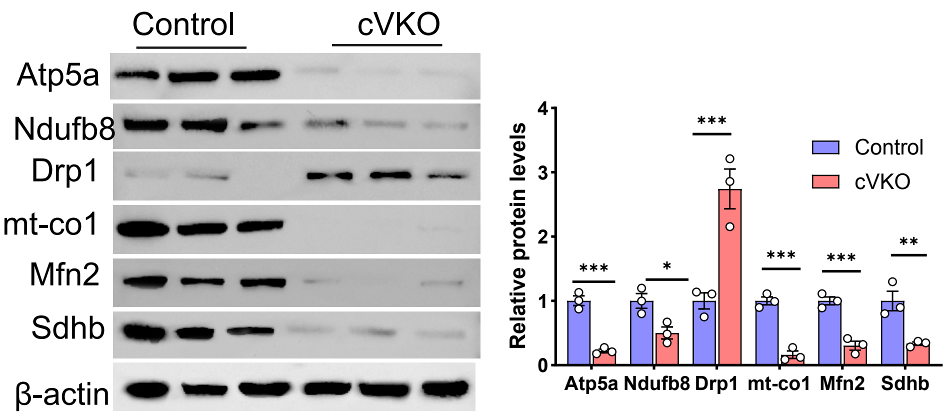
Control
cVKO
Atp5a
Ndufb8
Drp1
mt-co1
Mfn2
Sdhb
β-actin
Relative protein levels
4
3
2
1
0
***
*
***
***
***
**
Control
cVKO
Atp5a
Ndufb8
Drp1
mt-co1
Mfn2
Sdhb

S3

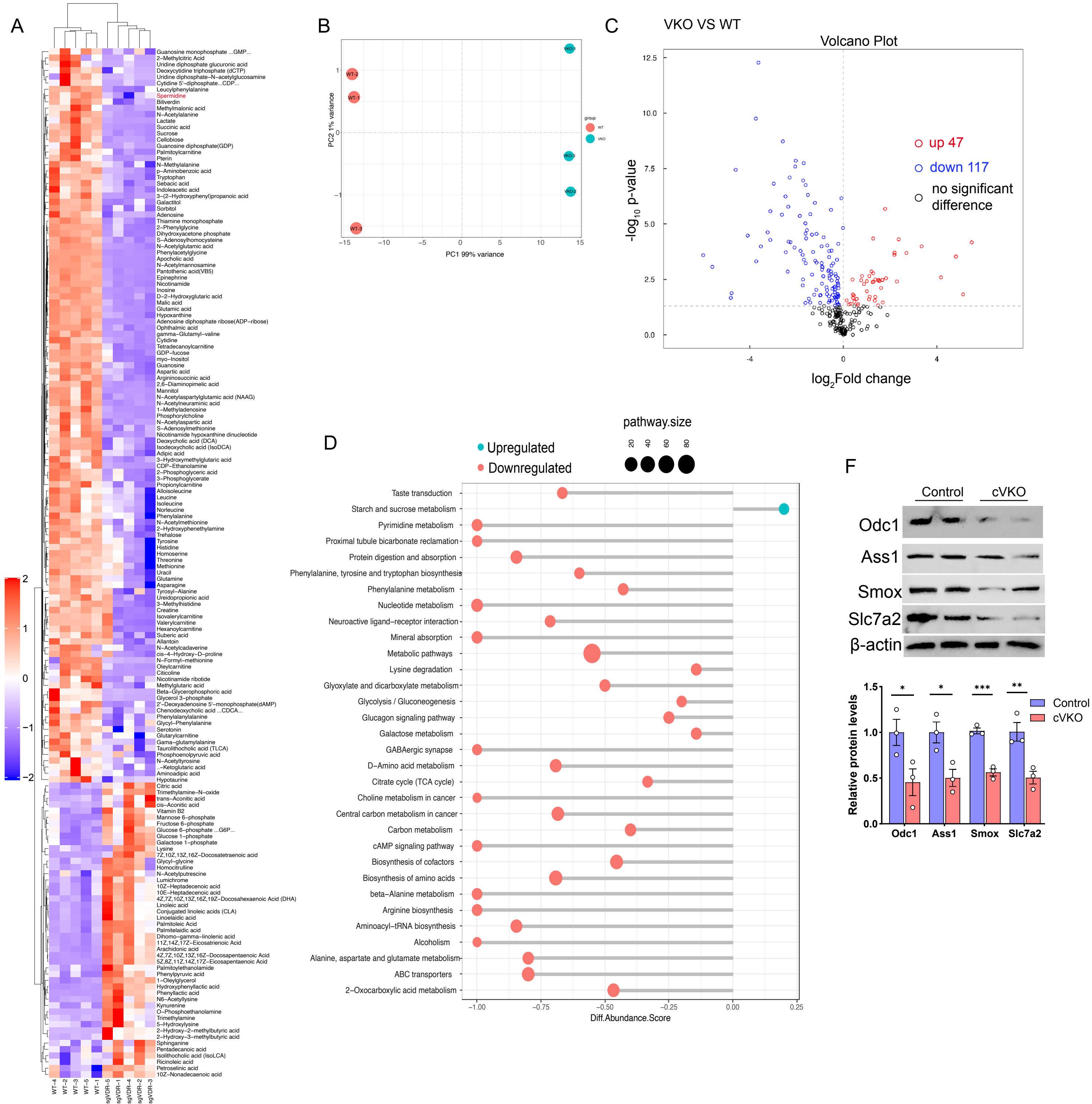

E

Matrix ID	Name	Score	Relative score	Sequence ID	Start	End	Strand	Predicted sequence
MA0693.2	MA0693.2.VDR	7.241532	0.869978	ODC1	936	943	+	CAAGTGCA
MA0693.2	MA0693.2.VDR	7.214498	0.8692072	ODC1	802	809	-	AGAGTTAA
MA0693.2	MA0693.2.VDR	6.747404	0.8558887	ODC1	1158	1165	+	TGAGTTCC
MA0693.2	MA0693.2.VDR	6.470487	0.84799284	ODC1	1241	1248	+	TGGCTTCA
MA0693.2	MA0693.2.VDR	5.7457595	0.8273283	ODC1	402	409	+	AAAGTTGA
MA0693.2	MA0693.2.VDR	5.429362	0.8183067	ODC1	1436	1443	+	CGGGATCA
MA0693.2	MA0693.2.VDR	5.222685	0.8124136	ODC1	37	44	-	AGGGTTCC
MA0693.2	MA0693.2.VDR	5.017806	0.8065718	ODC1	818	825	-	GGAGTACA

S4

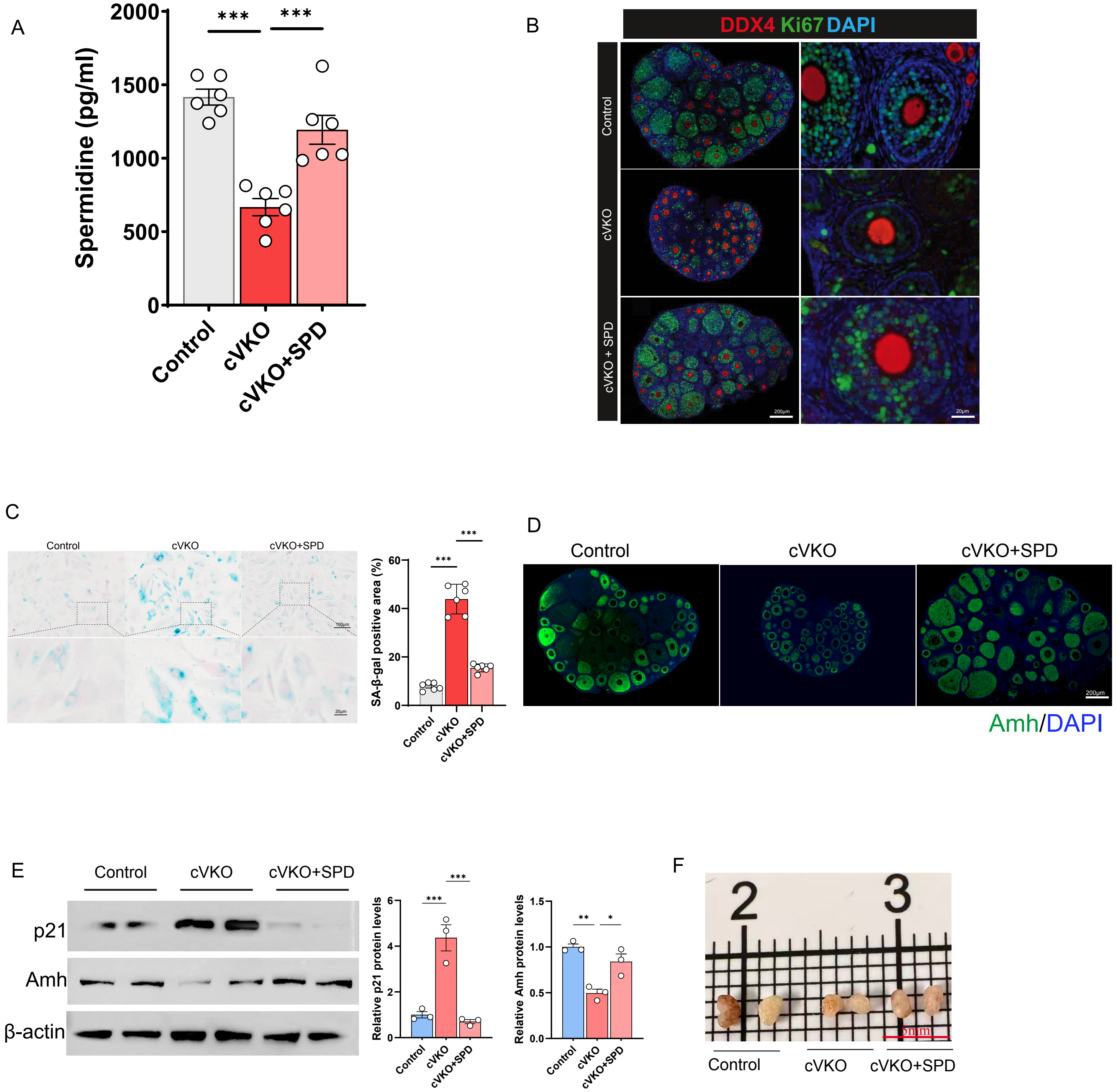
A
Spermidine (pg/ml)
2000
1500
1000
500
0
***
***
Control
cVKO
cVKO+SPD
B
DDX4 Ki67 DAPI
Control
cVKO
cVKO + SPD
C
Control
cVKO
cVKO+SPD
SA-β-gal positive area (%)
60
40
20
0
***
***
D
Control
cVKO
cVKO+SPD
Amh/DAPI
E
Control
cVKO
cVKO+SPD
p21
Amh
β-actin
Relative p21 protein levels
Relative Amh protein levels
***
**
*
F
2
3
Control
cVKO
cVKO+SPD

S5

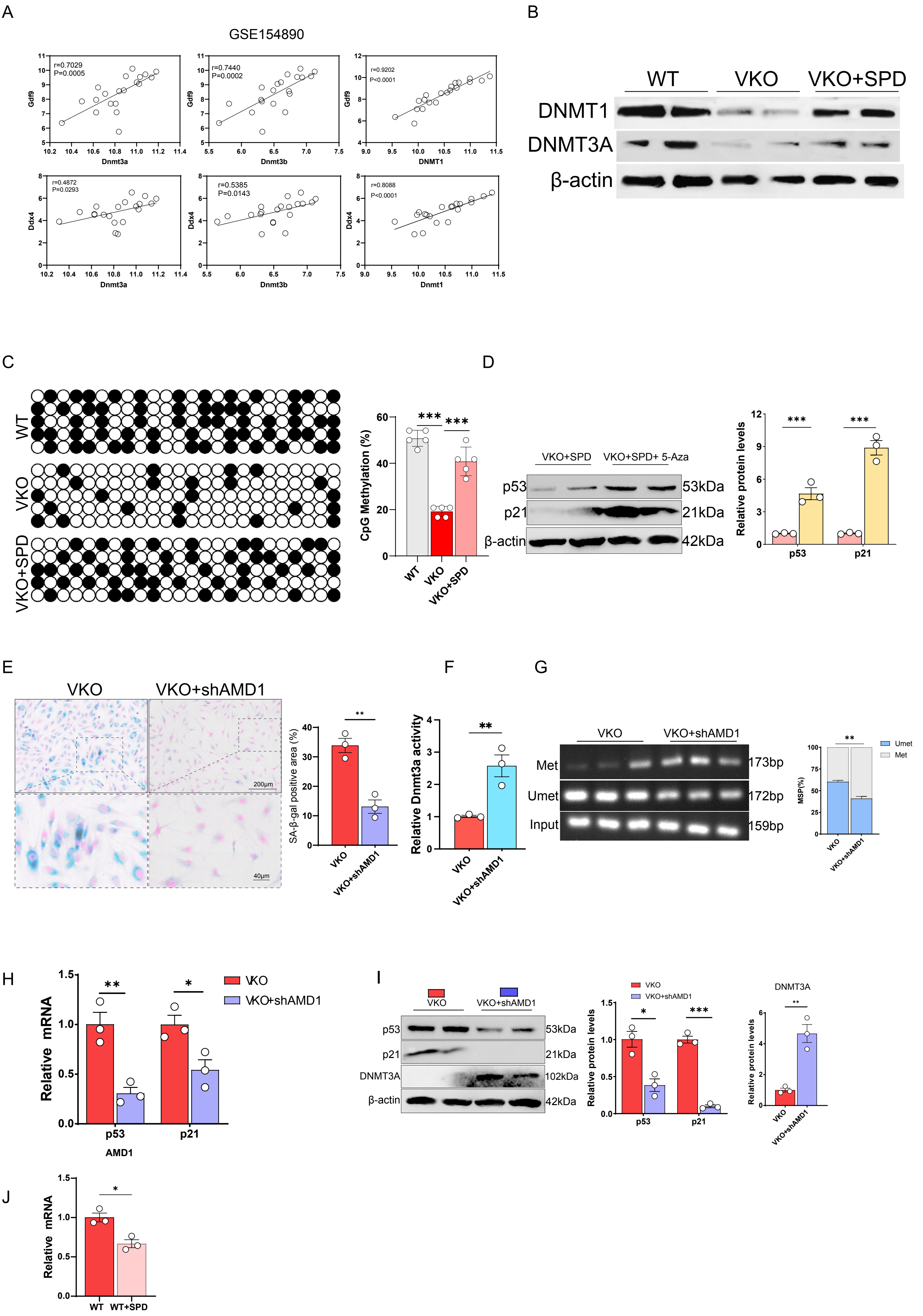
A
GSE154890
r=0.7029
P=0.0005
Gdf9
Dnmt3a
r=0.7440
P=0.0002
Dnmt3b
r=0.9202
P<0.0001
DNMT1
r=0.4872
P=0.0293
Ddx4
r=0.5385
P=0.0143
r=0.8088
P<0.0001
Dnmt1
B
WT
VKO
VKO+SPD
DNMT1
DNMT3A
β-actin
C
CpG Methylation (%)
D
VKO+SPD
VKO+SPD+ 5-Aza
p53
p21
β-actin
53kDa
21kDa
42kDa
Relative protein levels
E
VKO
VKO+shAMD1
200μm
40μm
SA-β-gal positive area (%)
F
Relative Dnmt3a activity
G
Met
Umet
Input
173bp
172bp
159bp
MSP(%)
H
Relative mRNA
AMD1
I
DNMT3A
102kDa
J

S6

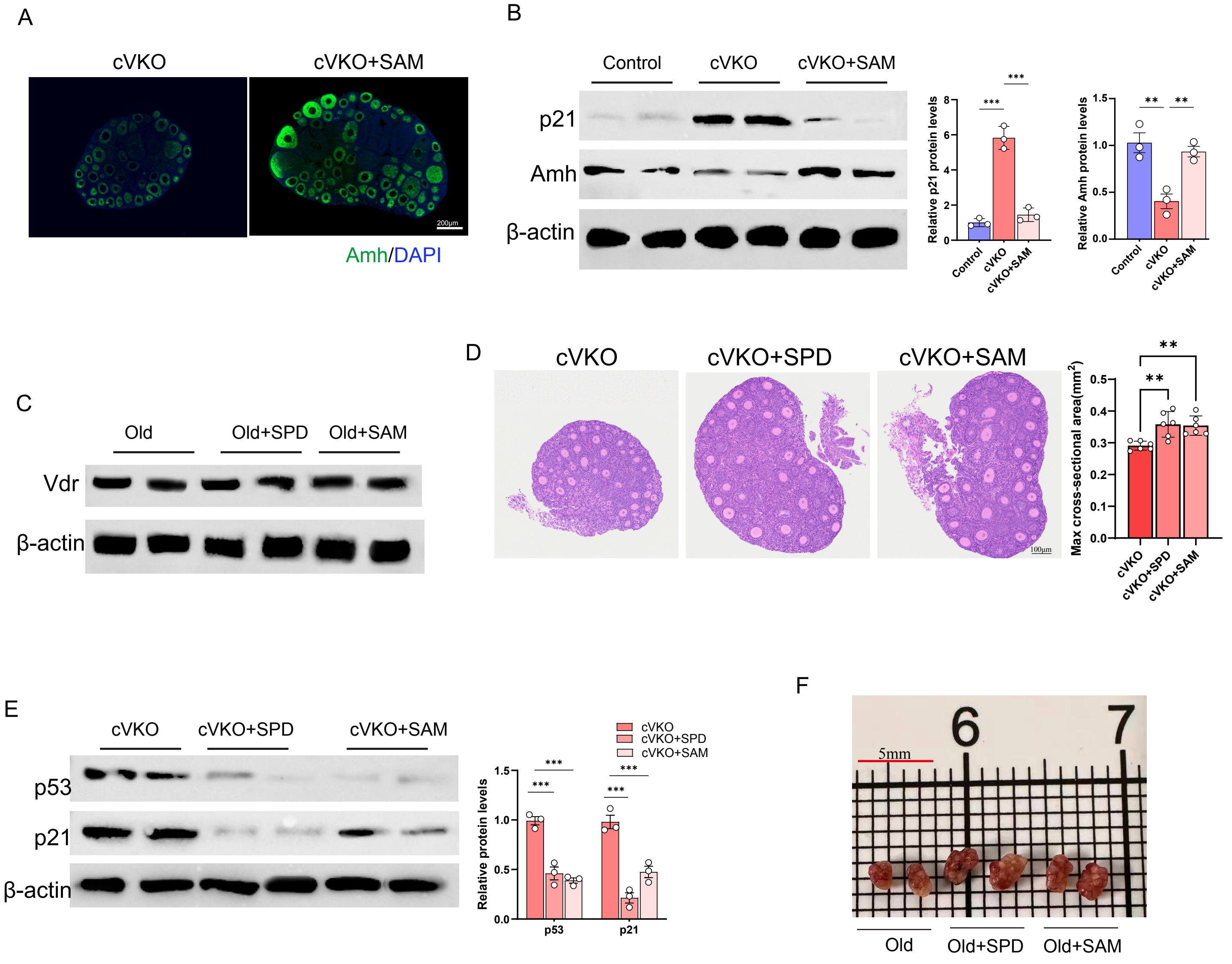
A
cVKO
cVKO+SAM
200μm
Amh/DAPI
B
Control
cVKO
cVKO+SAM
p21
Amh
β-actin
Relative p21 protein levels
Relative Amh protein levels
C
Old
Old+SPD
Old+SAM
Vdr
β-actin
D
cVKO
cVKO+SPD
cVKO+SAM
100μm
Max cross-sectional area(mm²)
E
cVKO
cVKO+SPD
cVKO+SAM
p53
p21
β-actin
Relative protein levels
F
5mm
6
7
Old
Old+SPD
Old+SAM
